# *Mycobacterium tuberculosis* IMPDH in Complexes with Substrates, Products and Antitubercular Compounds

**DOI:** 10.1371/journal.pone.0138976

**Published:** 2015-10-06

**Authors:** Magdalena Makowska-Grzyska, Youngchang Kim, Suresh Kumar Gorla, Yang Wei, Kavitha Mandapati, Minjia Zhang, Natalia Maltseva, Gyan Modi, Helena I. Boshoff, Minyi Gu, Courtney Aldrich, Gregory D. Cuny, Lizbeth Hedstrom, Andrzej Joachimiak

**Affiliations:** 1 Center for Structural Genomics of Infectious Diseases, University of Chicago, Chicago, IL, United States of America; 2 Structural Biology Center, Biosciences, Argonne National Laboratory, 9700 S Cass Ave. Argonne, IL, United States of America; 3 Department of Biology, Brandeis University, 415 South St. Waltham, MA, United States of America; 4 Tuberculosis Research Section, National Institute of Allergy and Infectious Diseases, Bethesda, MD, United States of America; 5 Center for Drug Design, Academic Health Center, University of Minnesota, 516 Delaware St. SE, Minneapolis, MN, United States of America; 6 Department of Pharmacological and Pharmaceutical Sciences, College of Pharmacy, University of Houston, 549A Science and Research Building 2, Houston, TX, United States of America; 7 Department of Chemistry, Brandeis University, 415 South St. Waltham, MA, United States of America; University of Padova, Medical School, ITALY

## Abstract

Tuberculosis (TB) remains a worldwide problem and the need for new drugs is increasingly more urgent with the emergence of multidrug- and extensively-drug resistant TB. Inosine 5’-monophosphate dehydrogenase 2 (IMPDH2) from *Mycobacterium tuberculosis* (*Mtb*) is an attractive drug target. The enzyme catalyzes the conversion of inosine 5’-monophosphate into xanthosine 5’-monophosphate with the concomitant reduction of NAD^+^ to NADH. This reaction controls flux into the guanine nucleotide pool. We report seventeen selective IMPDH inhibitors with antitubercular activity. The crystal structures of a deletion mutant of *Mtb*IMPDH2 in the apo form and in complex with the product XMP and substrate NAD^+^ are determined. We also report the structures of complexes with IMP and three structurally distinct inhibitors, including two with antitubercular activity. These structures will greatly facilitate the development of *Mtb*IMPDH2-targeted antibiotics.

## Introduction

Tuberculosis (TB) is a pandemic contagious infectious disease affecting nearly two billion people worldwide, with 5.7 million new cases reported in 2013 according to the World Health Organization [1]. The currently approved standard first-line treatment is long and requires combination therapy consisting of several antibiotics [2]. *Mycobacterium tuberculosis* (*Mtb*), the causative agent of most TB cases is a slow growing, remarkably successful pathogen capable of switching between different physiological states and adapting to disparate host environments [3,4]. The *Mtb* pathogen can persist in a quiescent state and survive for decades as a latent infection [5,6]. The increased prevalence of multidrug resistant (MDR) and extensively drug resistant (XDR) strains of *Mtb*, for which treatment options are very limited, demands the development of more effective antitubercular agents, ideally with novel mechanisms of action [6,7].

The design of antimetabolites that inhibit biosynthesis of essential metabolites within a cell is a classic approach for discovery of new antibiotics and chemotherapeutic agents. Indeed, one of the first reported TB drugs, *para*-aminosalicylic acid, was recently shown to disrupt folate biosynthesis [8], while the newest TB drug, bedaquiline exerts its activity through inhibition of ATP synthesis [9]. Inosine 5’-monophosphate dehydrogenase (IMPDH) lies at a key intersection of the purine biosynthesis pathway and represents an extremely attractive target since it controls flux of the guanine nucleotide pool. In many pathogens, guanine nucleotide levels are IMPDH-dependent and thus inhibition of IMPDH is a viable strategy for design of new chemotherapeutic agents [10]. The purine nucleotide biosynthetic pathway of *Mtb*, in common with other bacteria, contains three different enzymes from that of humans [11,12]: (1) *Mtb* contains both the folate dependent N^1^-(5-phospho-D-ribosyl)glycinamide (GAR) transformylase (PurN) and the formate/ATP utilizing formyl- N^1^-(5-phospho-D-ribosyl)glycinamide (FGAR) synthetase (PurT), whereas humans only contain GAR transformylase. (2) Humans use a type I 2-(formamido)-N^1^-(5-phospho-β-D-ribosyl)acetamidine (FAGM) synthetase composed only of PurL. Bacteria typically contain a type II FGAM synthetase, which is a complex of PurLQS. Orthologs for both PurL and PurQ have been identified in *Mtb*, as has a candidate PurS, which suggests that *Mtb* also utilizes the type II enzyme [11]. (3) Whereas humans convert 5-amino-1-(5-phospho-D- ribosyl)imidazole (AIR) directly to 5-amino-1-(5-phospho-D-ribosyl)imidazole-4-carboxylate (CAIR) via a class II AIR carboxylase (PurE class II), *Mtb* uses N^5^-carboxyaminoimidazole ribonucleotide (NCAIR) synthetase (PurK) to first convert AIR to NCAIR, then a class I AIR carboxylase converts NCAIR to CAIR. The remaining steps are common to both humans and *Mtb*. IMPDH catalyzes the NAD^+^-dependent conversion of inosine 5’-monophosphate (IMP) to xanthosine 5’-monophosphate (XMP), the first of the two step biosynthesis of guanosine 5’-monophosphate (GMP). GMP synthetase catalyzes the conversion of XMP to GMP in a reaction that also hydrolyzes glutamine to glutamate and ATP to AMP and pyrophosphate.

The *Mtb* H37Rv genome contains three genes with sequence similarity to bacterial IMPDH (*guaB1*, *guaB2*, and *guaB3*); the functions of *guaB1* and *guaB3* are poorly understood and only *guaB2* was shown to encode an active IMPDH [13],[14] (S1 Fig [54, 55]). Consistent with this finding, the *guaB2* gene is essential and cannot be rescued by the other orthologs [15]. The IMPDH activity of the *guaB2* gene product, *Mtb*IMPDH2, has been confirmed [14,16] and small molecule inhibitors have also been described [14,16,17].

**Fig 1:**
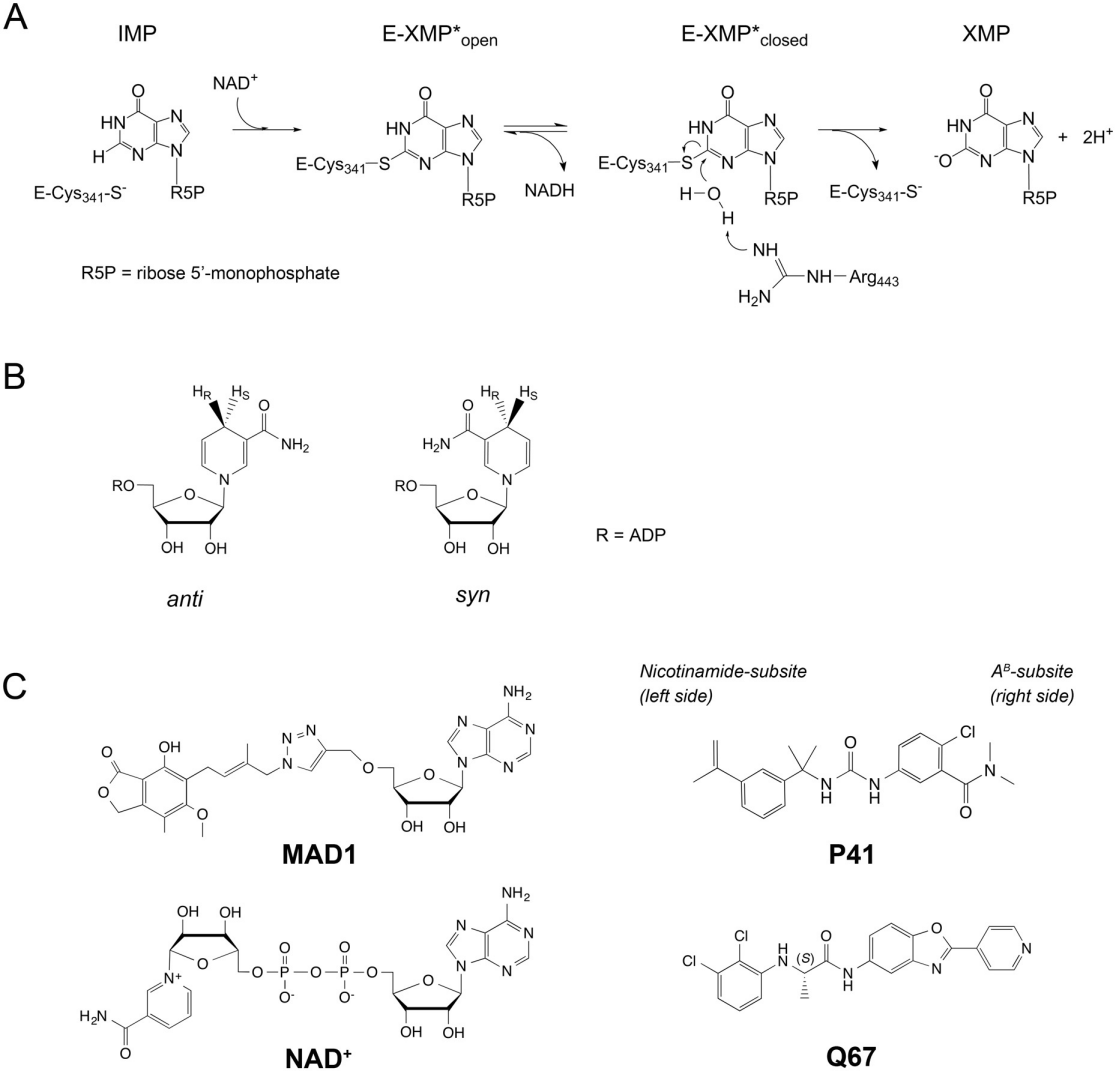
Mechanism and inhibitors of *Mtb*IMPDH2. (A) Reactions catalyzed by IMPDH. *Mtb*IMPDH2 numbering is shown. (B) The *anti*- and *syn*- conformations of nicotinamide ring in NADH. (C) *Cp*IMPDH inhibitors, for which crystal structures of *Mtb*IMPDH2•IMP•**I** were obtained. Portions of inhibitors that bind in the nicotinamide-subsite (left side) and the A^B^-subsite (right side) are indicated. Stereochemistry is denoted where applicable. NAD^+^ is shown for comparison.

The enzymatic mechanism of IMPDH has been extensively studied and consists two steps, a dehydrogenase and a hydrolase reaction (Fig 1A) [10,18]. Upon binding of IMP and NAD^+^ cofactor, a thioimidate enzyme-substrate adduct, E-XMP*, is formed via a covalent bond to the catalytic C341 (*Mtb*IMPDH2 numbering) with concurrent production of NADH. Hydride transfer occurs to the *pro-S* position with the cofactor in the *anti-*conformation (Fig 1B). The cofactor is released and an active site mobile flap moves into the NAD^+^ site and facilitates E-XMP* hydrolysis with a conserved R443 acting as a general base. Thus, the enzyme has two distinct conformations, an open form for the dehydrogenase reaction and a closed form for E-XMP* hydrolysis. IMPDH has multiple active site states (apoE, E•IMP, E•IMP•NAD^+^, E-XMP*•NADH adduct) that can be targeted for inhibitor design, and both the IMP/XMP and cofactor binding sites have been exploited for that purpose [10,18,19].

Human and bacterial IMPDHs differ in their affinity toward the NAD^+^ cofactor. While the nicotinamide portion of NAD^+^ binds similarly in human and *Vibrio cholerae* IMPDHs to preserve the interaction with IMP for hydride transfer, the interactions of the adenosine moiety vary dramatically. In the human type II enzyme (hIMPDH2), the adenine ring π/π stacks between a pair of aromatic residues within the same monomer as the IMP binding site (H253 and F282 in hIMPDH2; referred to as the A^E^-subsite; S1 Fig) and the cofactor maintains the extended conformation typically found in dehydrogenases [20]. In contrast, in *V*. *cholera* IMPDH (*Vc*IMPDH), the adenine ring binds to the adjacent monomer and interacts with a different set of residues (referred to as the A^B^-subsite; S1 Fig) and the cofactor assumes a rare compact conformation [21]. It also appears that adenine binding to this site is less constrained because two different conformations are observed for this moiety in two crystal structures of *Vc*IMPDH[21]. This provides critical information that the design of bacteria-selective inhibitors should focus on the A^B^-subsite. Importantly, compounds that bind with high affinity to this site should not bind to human IMPDHs.

Bacterial IMPDH-specific compounds were discovered in a high throughput screen for inhibitors of *Cryptosporidum parvum* IMPDH (*Cp*IMPDH). Although *C*. *parvum* is a protozoa, its IMPDH is a bacterial-like IMPDH [22]. Multiple potent chemotypes have been developed as inhibitors of this enzyme (designated as classes A, C, D, N, P, and Q, among others) [23–29]. These inhibitors target the A^B^-subsite and thus show high selectivity for bacterial IMPDHs. Moreover, structural studies of *Cp*IMPDH with representatives from two classes, **C64** and **Q21**, identified an “inhibitor minimal structural motif” (IMSM) consisting of A165 and Y358’ (prime denotes a residue from the adjacent monomer) that is required for compound binding [27,30–32]. Bacterial IMPDHs have high sequence similarities and the IMSM motif is found in many species, including *Mtb*IMPDH2 with A285/Y487’ in the corresponding positions. However, despite this sequence conservation, the structure–activity relationships (SAR) of a given inhibitor chemotype are not preserved between different bacterial IMPDHs, thus structure-based compound optimization toward a given IMPDH cannot be performed by simple knowledge-based prediction, but requires experimental verification [21,31,32].

Numerous efforts by several groups in academia and industry directed at characterization of either apo or ligand-bound *Mtb*IMPDH2 structures have been unsuccessful, likely due to the very low solubility of the recombinant *Mtb*IMPDH2 protein expressed in *E*. *coli*. We have overcome this major limitation by designing a variant that lacks the non-catalytic CBS subdomain (*Mtb*IMPDH2ΔCBS), greatly improving solubility [21] without impacting enzyme catalytic properties. Here we present the first crystal structures of *Mtb*IMPDH2ΔCBS, including the apoenzyme, the E•XMP•NAD^+^ complex, and complexes with IMP and three inhibitors, **MAD1**, **P41**, and **Q67** (Fig 1C). In addition, we report the SAR for *Mtb*IMPDH2ΔCBS inhibition along with antibacterial activity of several inhibitors from structurally distinct classes.

## Results

### Engineering IMPDH for inhibitor and structural studies

Deletion of IMPDH CBS domains facilitated crystallization of enzymes from several other organisms [21], therefore we constructed a variant of *Mtb*IMPDH2 wherein residues E126-R252 were replaced with a GG linker. The *Mtb*IMPDH2ΔCBS showed significantly improved solubility and crystallizability properties[21] with the steady state kinetic parameters comparable to those reported for the wild type enzyme (Table 1 [16]). Similar results were reported recently for deletion mutants of three other bacterial IMPDHs [21]. Notably, the values of *K*_m_ for both IMP and NAD^+^ for *Mtb*IMPDH2 are significantly higher than those of the human enzymes, illustrating the functional differences between bacterial and human IMPDHs (Table 1 [33–36]).

**Table 1:** *Mtb*IMPDH2ΔCBS kinetic parameters.

	*Mtb*IMPDH2ΔCBS	*Mtb*IMPDH2^a^	hIMPDH1	hIMPDH2
Km (IMP), μM	41 ± 4	78 ± 6	14–18^b^ ^,^ ^c^ ^,^ ^d^	4–9^b^ ^,^ ^c^ ^,^ ^e^
Km (NAD+), μM	580 ± 30	1005 ± 95	42–70^b^ ^,^ ^c^ ^,^ ^d^	6–32^b^ ^,^ ^c^ ^,^ ^e^
Kii (NAD+), mM	16 ± 1	5.0 ± 0.6	2.0 ± 0.6^d^	0.59 ± 0.02^e^
kcat, s–1	0.57 ± 0.01	0.53 ± 0.03	1.2–1.8^b^ ^,^ ^c^ ^,^ ^d^	0.4–1.4^b^ ^,^ ^c^ ^,^ ^e^

^a^Reference [16]

^b^Reference [33]

^c^Reference [34]

^d^Reference [35]

^e^Reference [36]

### Antitubercular activity of *Cp*IMPDH inhibitors

We tested 139 compounds developed in our *Cp*IMPDH inhibitor program for antitubercular activity [21,24,25,27–30,32,37,38], including compounds from the **A** benzotriazole (21), **C** benzimidazole (9), **D** phthalazinone (19), **P** urea (52) and **Q** benzoxazole (37) structural series (S1–S7 Tables). Most of these compounds were expected to be potent inhibitors of *Mtb*IMPDH2 based on their behavior versus *Cp*IMPDH and *Bacillus anthracis* IMPDH (*Ba*IMPDH; S1–S8 Tables).

**Fig 2:**
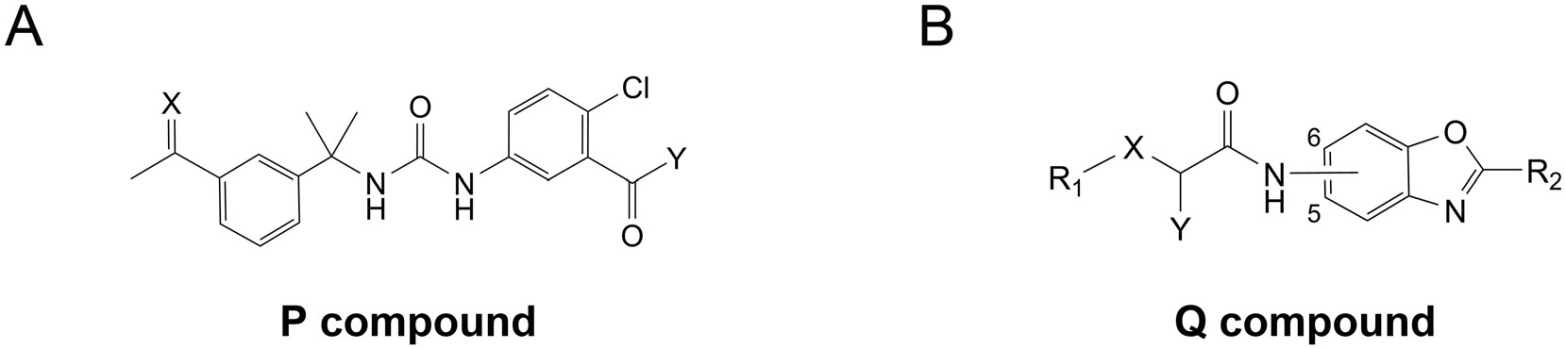
General structures of *Cp*IMPDH inhibitors from P and Q series. Modular structure of (A) P compounds and (B) Q compounds. Substituents X, Y, R_1_ and R_2_ are listed in Table 3. Designation “5” and “6” refers to the position in the benzoxazole ring of Q compounds.

Five **P** compounds and twelve **Q** compounds displayed significant activity against *Mtb* strain H37Rv in minimal BSA-free medium (MIC ≤20 μM, Fig 2, Tables 2 and 3). The compounds were somewhat less effective in BSA-supplemented rich media (Table 2). No active compounds were identified in the other structural series. The active compounds have significantly more polar surface area (average topological polar surface area (tPSA) 83 ± 18 Å^2^) than the inactive compounds (average tPSA = 64 ± 4 Å^2^, p < 0.001). The active and inactive compounds have similar hydrophobicity (average cLogP = 4.1 ± 0.8 for the active versus cLogP = 4.4 ± 1.3 for the inactive). All of the active compounds were potent inhibitors of *Mtb*IMPDH2ΔCBS, with values of *K*_*i*,*app*_ ranging from 13–2000 nM (Table 2). Notably, **P67** and **Q67** are the most potent inhibitors of *Mtb*IMPDH reported to date.

**Table 2:** Antitubercular activity of *Cp*IMPDH inhibitors. The values of *K*_*i*,*app*_ for inhibition of *Mtb*IMPDH2ΔCBS.

Cmpd	*K*_*i*_,_*app*_ ^a^ (nM)	cLogP	tPSA (Å^2^)	MIC in GAST media				MIC in 7H9 media			
				- Gua		+ Gua		- Gua		+ Gua	
				MIC (μM)	N	MIC (μM)	N	MIC (μM)	N	MIC (μM)	N
**P32**	158 ^b^	2.4	116.8	11.4 ± 5.3	4	≥40	3	27.2 ± 15.7	4	≥38	3
**P41**	17 ± 4	4.56	61.4	5.0 ± 3.1	4	≥40	3	33.3 ± 13.5	4	≥38	3
**P67**	13 ± 5	3.34	94.0	2.9 ± 0.4	3	≥40	3	15.7 ± 5.5	3	19 ± 6	3
**P146**	37 ±8	3.53	103.3	14.9 ± 10.0	2	>50	1	28.0 ± 9.0	2	44 ± 7	2
**P150**	35 ± 3	4.42	94.03	14.2 ± 5.0	2	>50	1	43.5 ± 6.0	2	63 ± 13	2
**Q9**	650 ± 140	3.59	72.3	17.5 ± 7.0	2	50	1	28.0 ± 19.2	3	≥37	2
**Q22**	2000 ± 60 ^c^	5.18	72.3	19.8 ± 14.9	3	25 ± 12	2	25.3 ± 8.3	4	50 ± 40	3
**Q27**	240 ± 40	4.21	72.3	15.2 ± 8.2	4	38 ±12	3	19.1 ± 13.3	4	60 ± 40	3
**Q33**	150 ± 50	3.68	81.5	5.3 ± 0.9	3	24 ± 16	3	10.4 ± 1.8	3	21 ± 4	3
**Q36**	76 ± 27 ^c^	4.85	72.3	9.4 ± 3.0	2	37	1	15.8 ± 5.5	3	28 ± 9	2
**Q42**	620 ± 70	2.97	90.7	17.3 ± 4.1	5	41 ± 6	4	22.0 ± 9.1	5	31 ± 7	4
**Q46**	130 ± 40	4.01	124.1	11.6 ± 5.7	4	38 ± 12	3	19.5 ± 4.1	4	44 ± 7	2
**Q49**	440 ± 40	3.61	63.1	12.7 ± 5.3	3	31 ± 19	2	21.1 ± 11.6	4	31 ± 6	3
**Q59**	40 ± 7	4.85	72.3	9.7 ± 4.1	4	26 ± 12	3	9.1 ± 3.1	4	25 ± 20	3
**Q60**	40 ± 16	5.02	75.1	14.5 ± 4.0	2	>50	1	14.5 ± 4.8	3	19 ± 0	2
**Q67**	14 ± 3	5.02	75.1	6.7 ± 3.0	2	50	1	11.8 ± 6.3	3	35 ± 15	2
**Q77**	100 ± 20	4.21	72.3	6.3 ± 3.0	2	37	1	11.0 ± 2.0	2	19 ± 0	2
**MAD1**	1580 ± 70 ^d^	1.57	209	100 ^e^	1	>100 ^e^	1	50	1	75	1

^a^. Average and standard deviation of three independent determinations unless otherwise noted.

^b^. n = 1.

^c^. n = 2.

^d^. *K*_*i*,*app*_ of 1500 nM was determined for inhibition of wild-type *Mtb*IMPDH2 reported in [16]

^*e*^. MIC determined after two weeks.

**Table 3:** Structures of *Cp*IMPDH inhibitors with antitubercular activity.

P compound			Q compound					
Cmpd	X	Y	Cmpd	R_1_	X	Y	Connection	R_2_
**P32**	NOH	NH_2_	**Q9**	Ph	O	CH_3_	5	4-Py
**P41**	CH_2_	N(CH_3_)_2_	**Q22**	2,4-di-ClPh	O	CH_3_	5	2-Py
**P67**	NOH	N(CH_3_)_2_	**Q27**	2-ClPh	O	CH_3_	5	4-Py
**P146**	NOH	4-morpholinyl	**Q33**	4-OCH_3_Ph	O	CH_3_	5	4-Py
**P150**	NOH	N(CH_2_CH_3_)_2_	**Q36**	2,3-di-ClPh	O	*(S)*-CH_3_	5	4-Py
			**Q42**	2,3-di-OCH_3_Ph	O	*(S)*-CH_3_	5	4-Py
			**Q46**	2-Cl,3-NO_2_Ph	O	*(S)*-CH_3_	5	4-Py
			**Q49**		Ph	*(S)*-CH_3_	5	4-Py
			**Q59**	2,3-di-ClPh	O	*(S)*-CH_3_	6	4-Py
			**Q60**	2,3-di-ClPh	NH	CH_3_	5	4-Py
			**Q67**	2,3-di-ClPh	NH	*(S)*-CH_3_	5	4-Py
			**Q77**	2-ClPh	O	*(S)*-CH_3_	5	4-Py

Several observations suggest that antitubercular activity results from inhibition of *Mtb*IMPDH2. First, *(S)-*isomers of the **Q** compounds inhibit bacterial IMPDHs, while the *(R)*- isomers are inactive [27]. The racemate **Q60** has approximately half the antitubercular activity as the *(S)*-isomer **Q67**, as expected if *Mtb*IMPDH2 is the target (S7 Table). The values of MIC for ten active compounds, including all the **P** compounds, increased by at least a factor of 4 in the presence of guanine. This rescue provides strong evidence for the on-target activity of the compounds due to inhibition of *Mtb*IMPDH2 in the bacteria. The values of MIC increased by lesser extents for the remaining compounds (**Q9**, **Q22**, **Q27**, **Q42**, **Q46**, **Q49**, **Q59**). These compounds may also engage another target. Lastly, antitubercular activity correlated roughly with the potency of *Mtb*IMPDH2ΔCBS inhibition (Table 2 and S3 Fig).

All five **P** compounds also displayed antibacterial activity against *B*. *anthracis* (S8 Table [37]). Of the eight **Q** compounds also tested against *B*. *anthracis*, only **Q67** displayed activity against both bacteria. While **P32** and **Q67** have similar activity against both bacteria, **P41** and **P67** are 5-7-fold more effective against *Mtb* and **P146** and **P150** are 15-28-fold more effective against *B*. *anthracis*. Differences in the SARs for enzyme inhibition cannot simply account for these differences in antibacterial activity (S8 Table and S2 Fig). Therefore, differences in cellular accumulation likely determine antibacterial spectrum. Interestingly, the compounds active against *B*. *anthracis* are significantly less hydrophobic than those active against *Mtb* (cLogP = 3.5 ± 0.5, p = 0.018; S8 Table [37]).

The five active **P** compounds contain a 3-carboxamido-4-chlorophenyl ring. Remarkably, the 4-chloro substituent is also found in 21 inactive **P** compounds, suggesting that the 3-carboxamido-4-chlorophenyl ring is critical for antitubercular activity. The 3-piperazinylcarbonyl-4-chloro analog **P94** is inactive, suggesting that the positive charge is deleterious (S5 Table). The inactive compounds include the alkene analog of **P32** (**P16**), the ketone analog of **P32** (**P25**) and methyloxime analog of **P67** (**P74**). **P16**, **P25** and **P74** are also expected to be good inhibitors of *Mtb*IMPDH2, which suggests that the oxime group confers an advantage for cellular accumulation.

All of the active **Q** compounds except **Q22** contain the 2-(pyrid-4-yl)benzoxazole group, which largely reflects the SAR of enzyme inhibition for *Cp*IMPDH and the consequential predominance of this group in the **Q** pool (70%). Nine of the active **Q** compounds contain a 2-chloro-substituted phenyl ring, and five of these are 2,3-dichloro substituted. **Q58** and **Q64** are the only two inactive compounds that contain 2,3-dichlorophenyl group. **Q58** is the 2-(thiazol-2-yl)benzoxazole analog of the active compound **Q36**, while **Q64** is the 2-(thiazol-5-yl)benzoxazole analog of the active compound **Q59**. These substitutions are expected to decrease the potency of enzyme inhibition by a factor of 4–20 based on *Cp*IMPDH and *Ba*IMPDH data (S8 Table). Therefore the 2,3-dichlorophenyl group is important for antitubercular activity in the benzoxazole scaffold.

### Mechanism of *Mtb*IMPDH2ΔCBS inhibition

Although the *Cp*IMPDH inhibitors all bind in the cofactor site, their mechanisms of inhibition can vary depending on their relative affinities for the E•IMP and E-XMP* complexes. Therefore we determined the mechanism of inhibition for the representative antitubercular compounds **P41** and **Q67**, as well as for compound **MAD1**, the first reported inhibitor of *Mtb*IMPDH2 [16] (Fig 1C);. **MAD1** is a third generation mycophenolic adenine nucleotide (MAD) inhibitor [16,39,40] that bears the closest resemblance to NAD^+^ (Fig 1). All three compounds are uncompetitive inhibitors with respect to IMP as expected, implying interactions with the base of IMP are critical for binding (S9 Table). The mechanism of inhibition with respect to NAD^+^ varies among the three compounds (S9 Table). **MAD1** is an uncompetitive inhibitor, suggesting that this compound has a strong preference for the E-XMP* intermediate. **P41** is a noncompetitive inhibitor, indicating this compound has similar affinity to both E•IMP and E-XMP*. **Q67** is a competitive inhibitor versus NAD^+^, suggesting that it has a strong preference for E•IMP.

### Overall structure

Five high resolution crystal structures were obtained for *Mtb*IMPDH2ΔCBS (Table 4). These include the apo form, the XMP•NAD^+^ complex, and three IMP•inhibitor complexes (E•IMP•**I**) with **MAD1**, **P41**, and **Q67**. The structure of the tertiary complex of *Mtb*IMPDH2ΔCBS with XMP and NAD^+^ was obtained by soaking crystals containing the *Mtb*IMPDH2ΔCBS•IMP complex with 200 mM NAD^+^. Structures of protein-inhibitor complexes were obtained by co-crystallization with IMP and inhibitor. In all five structures, the first 26 or 27 N-terminal residues are disordered in every protein chain. An approximately 20-residue portion of the active site flap is also disordered, as observed for the majority of the IMPDH structures reported to date.

**Table 4:** Data collection and refinement statistics.

	*Mtb*IMPDH2ΔCBS	*Mtb*IMPDH2ΔCBS•IMP•MAD1	*Mtb*IMPDH2ΔCBS•IMP•P41	*Mtb*IMPDH2ΔCBS•IMP•Q67	*Mtb*IMPDH2ΔCBS•IMP•NAD^+^
***Data collection***					
Space group	*P*1	*I*4	*I*4	*I*4	*I*4
Cell dimensions					
*a*, *b*, *c* (Å)	75.22, 75.23, 75.28	88.23, 88.23, 84.63	87.94, 87.94, 84.76	88.25, 88.25, 84.27	88.15, 88.15, 85.51
*α*, *β*, *γ* (°)	108.3, 108.3, 111.9				
Protein molecules/ASU	4	1	1	1	1
Temperature (K)	100	100	100	100	100
Radiation source	APS, 19-ID	APS, 19-ID	APS, 19-ID	APS, 19-ID	APS, 19-ID
Wavelength (Å)	0.97918	0.97899	0.97899	0.97899	0.97918
Resolution (Å)^a^	35.73–1.70 (1.73–1.70)	35.76–1.90 (1.93–1.90)	35.67–2.00 (2.03–2.00)	30.47–1.76 (1.79–1.76)	35.80–1.60 (1.63–1.60)
Unique reflections	129346 (3628)	24204 (779)	21407 (938)	31906 (1555)	42708 (1975)
*R*_merge_ ^b^	0.056 (0.330)	0.105 (0.326)	0.130 (0.762)	0.089 (0.644)	0.075 (0.805)
〈*I*〉/〈σ*I*〉	17.2(2.6)	11.7(2.1)	8.2(1.8)	10.6(2.0)	29.0(1.81)
Completeness (%)	91.4 (51.5)	94.5 (61.0)	98.0 (86.1)	99.7 (97.5)	99.5 (93.1)
Redundancy	2.2 (1.8)	4.2 (2.5)	3.9 (2.1)	4.4 (2.7)	5.8 (4.0)
***Refinement***					
Resolution (Å) ^*a*^	35.73–1.70 (1.71–1.70)	35.76–1.90(1.96–1.90)	35.67–2.00 (2.09–2.00)	30.47–1.76 (1.81–1.76)	35.80–1.60 (1.64–1.60)
Reflections: work/test set ^*a*^	122867/6432 (2097/107)	23811/1272 (2103/117)	20300/1090 (2219/109)	30282/1619 (2437/130)	40394/2185 (2343/103)
*R*_work_/*R*_free_ ^*c*^ ^,^ ^*a*^	0.151/0.183 (0.242/0.269)	0.143/0.189 (0.219/0.286)	0.178/0.221 (0.282/0.338)	0.153/0.179 (0.239/0.250)	0.160/0.191 (0.231/0.286)
No. of atoms: protein/ligands^d^/water	11793/170/793	2585/84/169	2527/51/118	2640/64/188	2386/68/161
Average *B* factor (Å^2^): protein/ligands/water	28.8/35.7/45.8	27.4/37.9/34.2	52.2/40.7/48.8	31.0/29.5/39.1	30.5/28.8/35.2
Bond lengths (Å)	0.010	0.010	0.007	0.007	0.009
Bond angles (°)	1.282	1.380	1.209	1.169	1.325
Most favored	98.4	97.5	97.7	98.6	98.2
Outliers	0.0	0.0	0.28	0.0	0.0
PDB accession code	4ZQR	4ZQP	4ZQN	4ZQO	4ZQM
***Crystallization conditions***	0.1M sodium/potassium phosphate pH 6.2, 25% 1,2-propandiol, 10% glycerol, 16°C	0.1M sodium/potassium phosphate pH 6.2, 25% 1,2-propandiol, 10% glycerol, 16°C	0.3M magnesium formate, 0.1M Tris pH 8.5, 16°C	0.1M sodium/potassium phosphate pH 6.2, 25% 1,2-propandiol, 10% glycerol, 16°C	0.4M magnesium formate, 0.1M Tris-HCl pH 8.5, 20% sucrose, 16°C, soaked with 200 mM NAD

ASU, Asymmetric Unit,

^*a*^Values in parentheses correspond to the highest-resolution shell.

^*b*^ *R*_*merge*_ = Σ_*hkΣi*_|*I*_*i*_(*hkl*)– 〈*I(hkl)*〉|/Σ_*hkl*_Σ_*i*_|〈*I*_*i*_ *(hkl)*〉|, where *I*_*i*_(hkl) is the intensity for the *i*th measurement of an equivalent reflection with indices *h*, *k*, and *l*.

^*c*^ *R*_*work*_ = Σ_*hkl*_||*F*_*obs*_|—|*F*_*calc*_||/ Σ_*hkl*_ |*F*_*obs*_|, where *F*_*obs*_ and *F*_*calc*_ are observed and calculated structure factors, respectively. *R*_*free*_ is calculated analogously for the test reflections, which were randomly selected and excluded from the refinement.

^*d*^Ligands include all atoms excluding protein and water atoms.

### Structure of Apo *Mtb*IMPDH2ΔCBS

**Fig 3:**
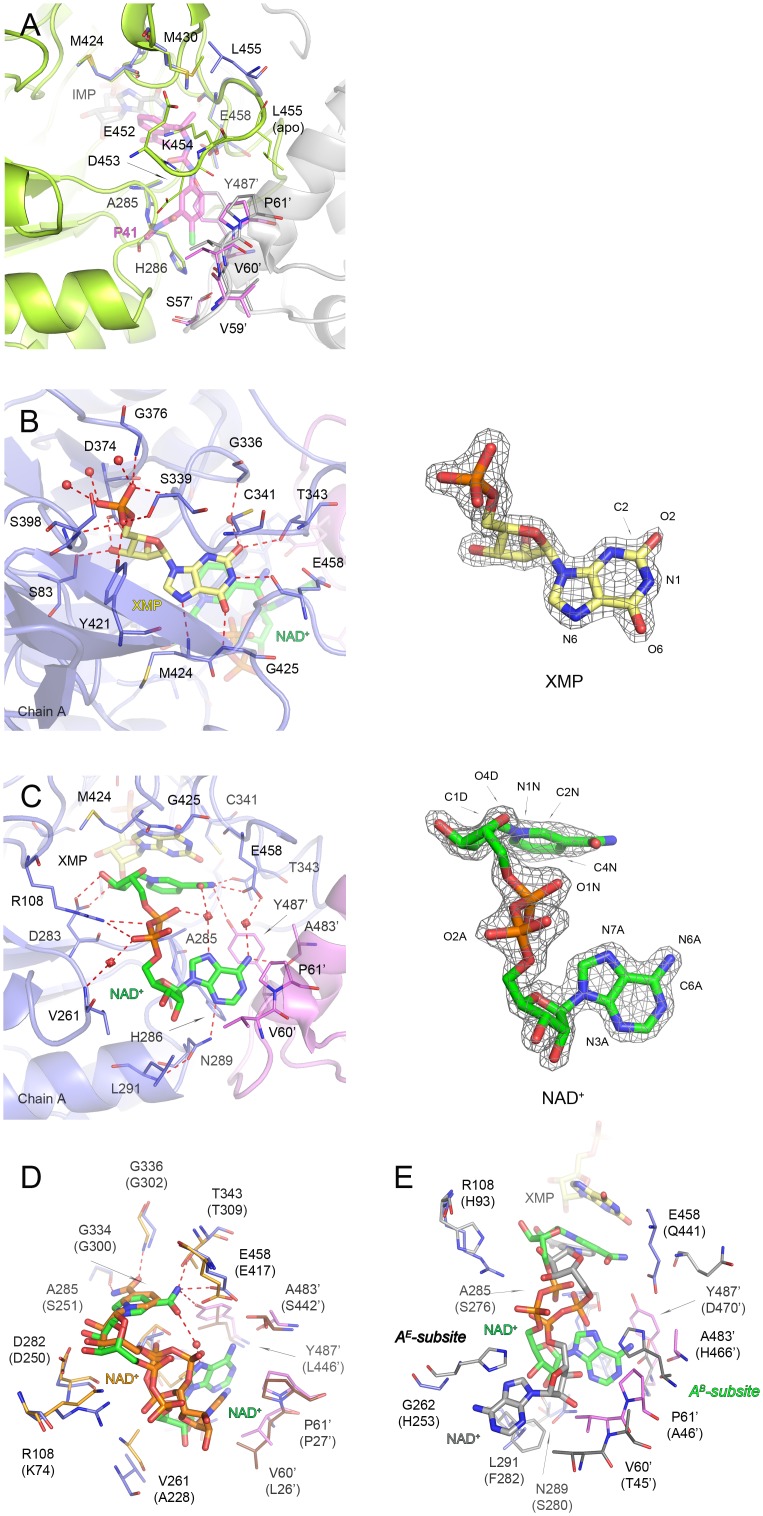
Active site flap in apo *Mtb*IMPDH2ΔCBS and cofactor orientation in *Mtb*IMPDH2ΔCBS•XMP•NAD^+^ complex. (A) Overlay of apo *Mtb*IMPDH2ΔCBS and *Mtb*IMPDH2ΔCBS•IMP•**P41** structures with a flap residue K454 in the apo form clashing with the linker position of **P41**, indicating that these two elements occupy the same space in the active site. For *Mtb*IMPDH2ΔCBS•IMP•**P41**, only residues (lines) and **P41** (sticks) are shown; color code for *Mtb*IMPDH2ΔCBS•IMP•**P41** as in Fig 5A. For the apo structure, chains A (lime) and C (gray) are shown in a cartoon representation and residues corresponding to these involved in inhibitor binding are shown as lines. A prime denotes a residue from the adjacent monomer. (B) Top view of the active site showing XMP interactions. Chain A (slate blue) and symmetry-generated adjacent chain (violet) are shown. Residues are represented as lines. XMP (pale yellow) and NAD^+^ (green) are shown as sticks. (C) Side view of the active site detailing NAD^+^ binding. Color code and depiction as in panel (B). For panels (B) and (C) 2m*Fo*-D*Fc* electron density maps contoured at the 2 σ level for XMP (pale yellow) and 1.5 σ level for NAD^+^ (green) are shown on the right. Atoms discussed in text are labeled. (D) Cofactor position in superimposed structures *Mtb*IMPDH2ΔCBS•XMP•NAD^+^ and *Vc*IMPDHΔCBS•XMP•NAD^+^. Only ligands (depicted as sticks) and the interacting residues (represented as lines) are shown. Residues are labeled according to *Mtb*IMPDH2 numbering with *Vc*IMPDH numbering in parentheses. Color code is as follows: for the *Mtb* structure as in panel (A); for the *V*c structure: chain A (light orange), symmetry-generated adjacent chain (brown), NAD^+^ (orange), XMP and selected hydrogen bonds are omitted for clarity. (E) Overlay of the cofactor position in *Mtb*IMPDH2ΔCBS•XMP•NAD^+^ and the ternary complex of hIMPDH2 with NAD^+^ and substrate analog, CPR (hIMPDH2•CPR•NAD^+^; PDB code 1NFB). Residues are labeled according to *Mtb*IMPDH2 numbering with hIMPDH2 numbering in parentheses. Color code is as follows: for the *Mtb* structure as in panel (B); for the human structure: chain A (light gray), symmetry-generated adjacent chain (dark gray), NAD^+^ (gray), CPR is omitted for clarity. Localization of the eukaryotic A^E^-subsite and the bacterial A^B^-subsite is indicated. For all panels (where applicable): a prime denotes a residue from the adjacent monomer. Water molecules are shown as red spheres. Hydrogen bonds are depicted as red dashed lines.

The apo structure contains four polypeptide chains in the asymmetric unit (rmsds for Cα atoms of chain A versus other three are from 0.10 to 0.12 Å). A potassium ion is bound between subunits, interacting with six main chain carbonyls, three from the loop containing active site C341 (G336, G338, and C341) and three from the C-terminal portion of the adjacent subunit (E551’, S512’, and H513’). This coordination is very similar to that observed in previously reported structures [21,41,42]. A phosphate ion originating from the crystallization buffer binds in the phosphate site of IMP, anchored by hydrogen bonding interactions with the backbone nitrogen atoms of S339, G376, G397 and S398 and the side chains of S339 and Y421. This interaction network is further supplemented by water-mediated contacts. Interestingly, K454 (residue in the active site flap) occupies the position where the linker portions of **P41** and **Q67** bind (see below, Fig 3A). This residue must move out of the active site when an inhibitor binds. Thus the conformational dynamics of the active site flap may contribute to the differences in inhibitor affinities among IMPDHs from different sources.

### *Mtb*IMPDH2ΔCBS complex with XMP and NAD^+^

The 1.60 Å resolution crystal structure of the product/cofactor complex contains one protein chain per asymmetric unit. Although no potassium was present in the crystallization buffer and this ion is not found in the complex, the high quality electron density maps clearly show that the product XMP is present in the active site (Fig 3B). This indicates that the enzyme in the crystal is catalytically competent. XMP is in essentially the same orientation as observed previously in IMPDH from *V*. *cholerae* (PDB id 4X3Z) [21]. The xanthine ring contacts C341, T343, M424, G425, and E458 and also has water-mediated interactions with the main chain nitrogen atom of G336 (Fig 3B). The phosphate group of XMP interacts with S339, G376, G397, S398, and Y421, and the sugar moiety forms hydrogen bonds with D374. These residues are highly conserved in all IMPDHs, with the exception of E458, which is replaced by glutamine in eukaryotic IMPDHs.

As observed for *Vc*IMPDHΔCBS•XMP•NAD^+^ [21], the NAD^+^ adenosine moiety binds in the A^B^-subsite located at the subunit interface and interacts with residues from both monomers (Fig 3C and 3D). For comparison, binding of adenosine in the A^E^-subsite of the hIMPDH2 complex with NAD^+^ and a substrate analog, CPR (hIMPDH2•CPR•NAD^+^; PDB code 1NFB) is shown in Fig 3E. In *Mtb*IMPDH2ΔCBS•XMP•NAD^+^ the adenine ring is in the *anti*-orientation with respect to the sugar ring. The adenine N3A atom contacts the side chain of N289, whereas the N6A amine makes one hydrogen bond with the carbonyl group of A483’ and one water-mediated contact with the side chain of E458. In addition, one side of the adenine ring participates in van der Waals interactions involving V60’ and P61’ and the other side π/π stacks with H286 in an edge-to-face orientation (Fig 3C). The same set of interactions is present in *Vc*IMPDHΔCBS•XMP•NAD^+^, although *Vc*IMPDH has S256 in place of N289, L26’ instead of V60’ and the π/π stacking with the conserved histidine residue occurs in a face-to-face orientation. The N6A amine also makes a NH_2_/π interaction with the side chain of Y487’ (part of the ISMS) in *Mtb*IMPDH2ΔCBS. This contact is not present in *Vc*IMPDHΔCBS•XMP•NAD^+^ because *Vc*IMPDH has L446’ in place Y487’ (Fig 3D). It is also important to note the adenosine portion in *Mtb*IMPDH2ΔCBS•XMP•NAD^+^ is positioned deeper into the binding pocket than in *Vc*IMPDHΔCBS•XMP•NAD^+^ and the cofactor is in a more compact orientation (C6A-C2N distance of 9.16 Å in *Mtb*IMPDH2ΔCBS•XMP•NAD^+^ versus 11.41 ± 0.11 Å in *Vc*IMPDHΔCBS•XMP•NAD^+^ [21,43]). Interestingly, the position of the adenine group in *Mtb*IMPDH2ΔCBS•XMP•NAD^+^ is more similar to the *Vc*IMPDHΔCBS structure with NAD^+^ and a mixture of IMP and a covalent intermediate (*Vc*IMPDHΔCBS•IMP•NAD^+^; PDB id 4QNE [21]). The cofactor in *Vc*IMPDHΔCBS•IMP•NAD^+^ has similarly compact conformation (C6A-C2N distance of 9.42 ± 0.10 Å), with the adenine ring in an *anti*-conformation. Perhaps NAD^+^ changes orientation during the catalytic cycle prior to its dissociation from the active site, as suggested for *Vc*IMPDH [21].

The interactions of the NAD^+^ pyrophosphate are similar to those in previously reported cofactor structures. R108 makes two direct hydrogen bonds to O3 and O2A of the phosphate moiety and the same phosphate group makes one direct hydrogen bond to the amido group of A285 (part of the IMSM) and water-mediated bonds to the main chain amido and carbonyl groups of V261. The corresponding interactions, except for R108-O3/O2A, are also found in *Vc*IMPDHΔCBS•XMP•NAD^+^ [21].

The nicotinamide ring of NAD^+^ stacks against the xanthine ring of XMP and the nicotinamide ribose is anchored via hydrogen bonds between the hydroxyl groups and the conserved D283, as observed previously in other IMPDHs (Fig 3C). However, the orientation of the nicotinamide portion is opposite from that observed in other cofactor complexes. In *Vc*IMPDHΔCBS•XMP•NAD^+^, the nicotinamide ring is in the *anti*-conformation (Fig 3D) and the carboxamide group makes hydrogen bonds with two conserved glycine residues [21,44]. This conformation is consistent with hydride transfer to the *pro-S* position of NAD^+^ [45,46]. However, the nicotinamide ring flips ~180° and is in a *syn*-conformation in *Mtb*IMPDH2ΔCBS•XMP•NAD^+^ (with χ_N_ torsion angle of 5.7°; Figs 1B and 3C). The carboxamide moiety makes direct hydrogen bonds with side chains of T343, E458, and Y487’ (which is a part of the IMSM) and a water-mediated interaction with the O1N atom of the phosphate group and the N7A atom of adenine (Fig 3C and 3D). The carboxamide group also makes van der Waals contacts with A285 (also a part of the IMSM). As a result of the *syn*-conformation, the *pro-R* side of the nicotinamide ring now faces IMP with its C4N carbon aligned with the C2 of the IMP hypoxanthine ring (C4N-C2 distance of 3.28 Å).

We characterized the reaction of ^2^H-IMP and NAD^+^ to determine if the stereochemistry of hydride transfer catalyzed by *Mtb*IMPDHΔCBS was *pro-R* as suggested by the crystal structure. As reported previously [45,46], hIMPDH2 transferred deuterium to the *pro-S* position of NAD^+^ (S4 Fig). The deuterium also transferred to the *pro-S* position of NAD^+^ in the *Mtb*IMPDH2ΔCBS catalyzed reaction (S4 Fig). These observations indicate that the orientation of NAD^+^ observed in the XMP complex is not competent for hydride transfer and may reflect cofactor conformation stabilized by the IMSM.

### Inhibitor binding

**Fig 4:**
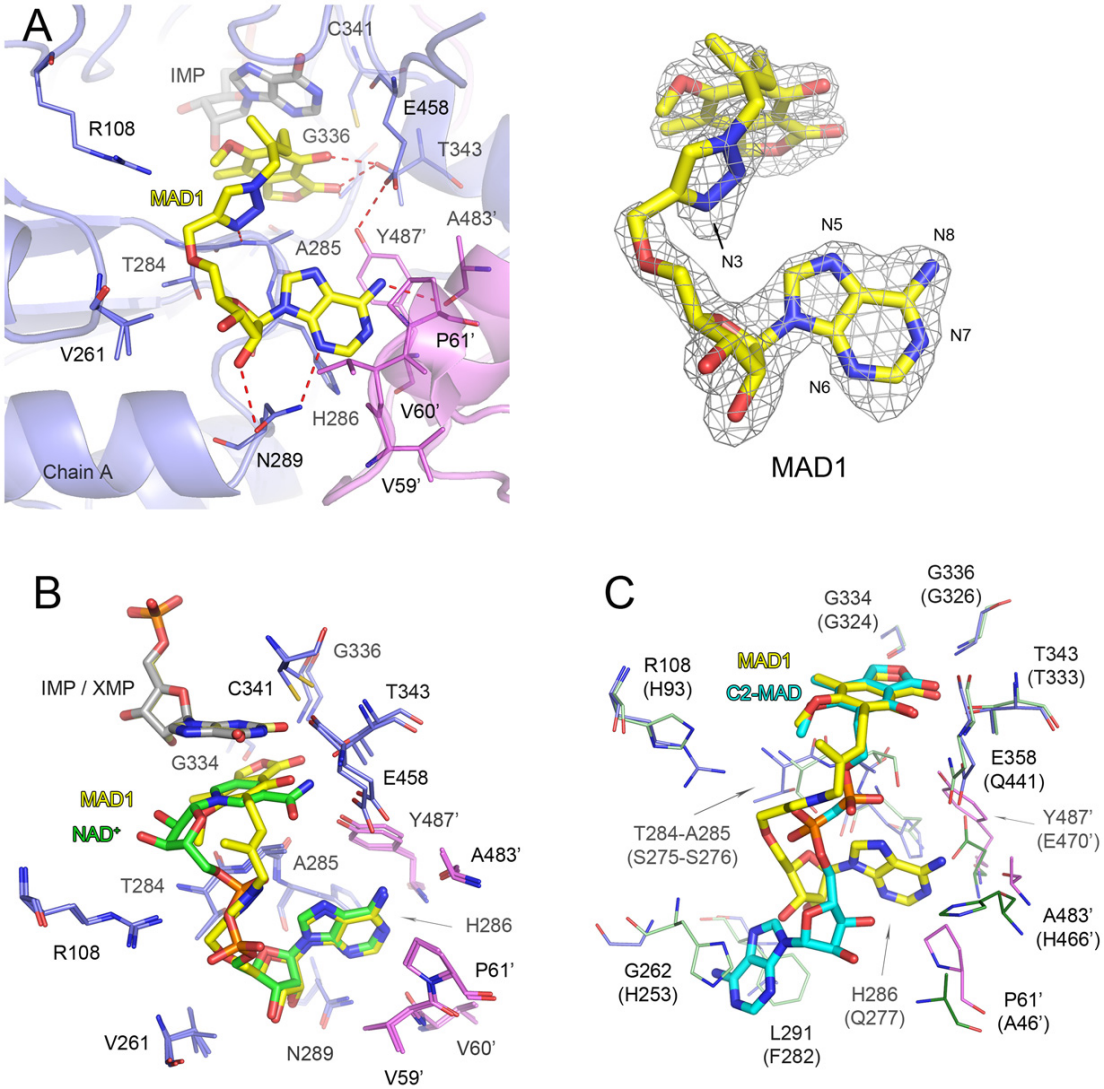
Binding of mycophenolic adenine nucleotide derivative MAD1 in *Mtb*IMPDH2ΔCBS complex. (A) *Mtb*IMPDH2ΔCBS•IMP•**MAD1** complex. Chain A (slate) and symmetry-generated adjacent chain (violet) are shown in a cartoon representation. Residues involved in inhibitor binding are shown as lines. A prime denotes a residue from the adjacent monomer. Molecules of IMP (light gray) and **MAD1** (yellow) are shown as sticks. Hydrogen bonds are shown as red dashed lines. 2m*F*_*o*_ –D*F*_*c*_ electron density map contoured at the 1 σ level for **MAD1** is shown on the right. Atoms discussed in text are labeled. (B) Overlay of *Mtb*IMPDH2ΔCBS•IMP•**MAD1** and *Mtb*IMPDH2ΔCBS•XMP•NAD^+^. Only the ligands (depicted as sticks) and the interacting residues (represented as lines) are shown. Color code for residues as in panel (A) and Fig 3, IMP (gray), **MAD1** (yellow), XMP (pale yellow), NAD^+^ (green). (C) Distinctive binding mode of MAD derivatives in bacterial and human IMPDHs. Overlay of *Mtb*IMPDH2ΔCBS•IMP•**MAD1** and hIMPDH2•RVP•C2-MAD. Only the inhibitors (depicted as sticks) and the interacting residues (represented as lines) are shown. Residues are labeled according to *Mtb*IMPDH2ΔCBS numbering with hIMPDH2 numbering in parenthesis. IMP and RVP are omitted for clarity. Color code: for *Mtb*IMPDH2ΔCBS•IMP•**MAD1** as in panel (A); for hIMPDH2, chain A (pale green), symmetry-generated adjacent chain (dark green), **C2-MAD** (teal).

**Fig 5:**
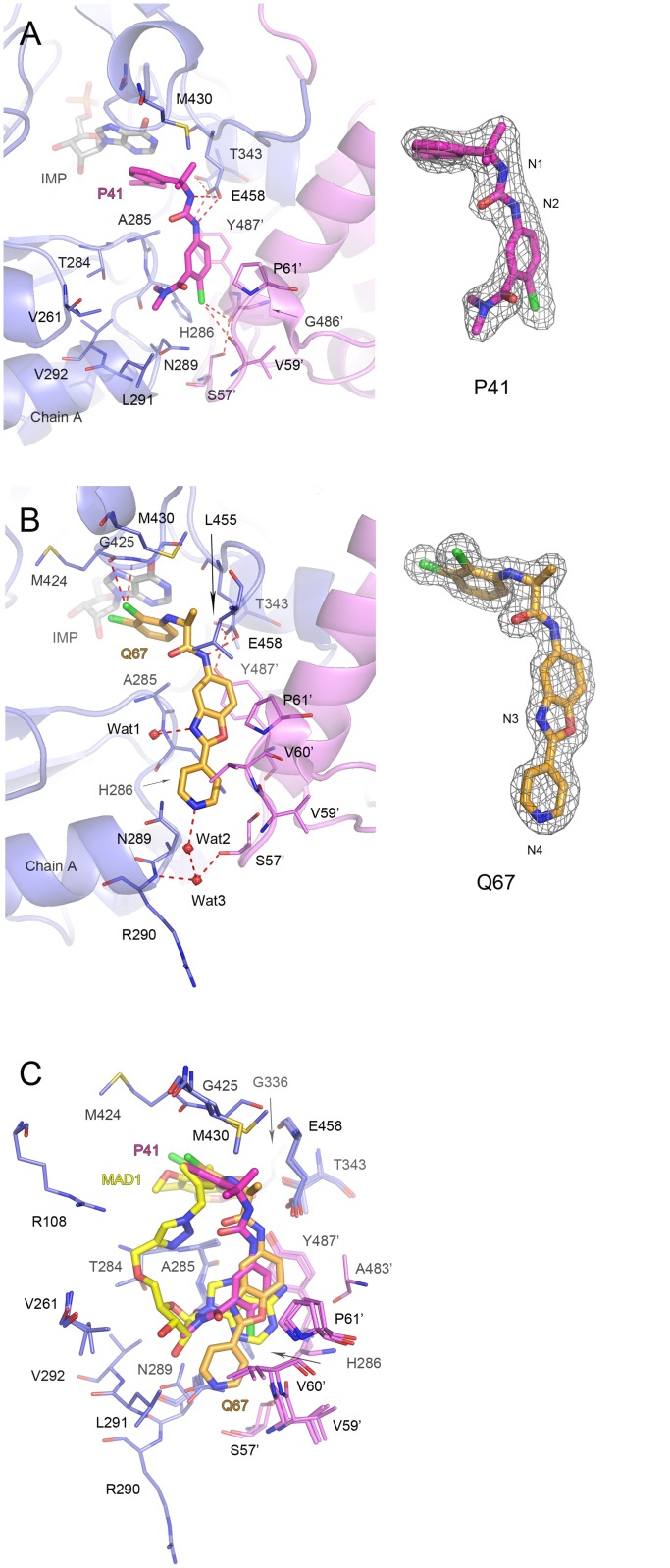
Binding of *Cp*IMPDH-selective inhibitors P41 and Q67. (A) *Mtb*IMPDH2ΔCBS•IMP•**P41** complex. (B) *Mtb*IMPDH2ΔCBS•IMP•**Q67** complex. Chain A (slate) and symmetry-generated adjacent chain (violet) are shown in a cartoon representation. Residues are shown as lines. A prime denotes a residue from the adjacent monomer. Molecules of IMP (light gray), **P41** (magenta), and **Q67** (orange) are shown as sticks. Water molecules are depicted as red spheres. Potential hydrogen and halogen bonds are shown as red dashed lines. For panels A and B 2m*F*_*o*_ –D*F*_*c*_ electron density map contoured at the 1 σ level for each inhibitor is shown on the right. Atoms discussed in text are labeled. (C) Overlay of three *Mtb*IMPDH2ΔCBS inhibitor complexes. IMP is omitted for clarity. Color designations as follows: for **MAD1** as in Fig 4A; for **P41** and **Q67** as in panels (A) and (B), respectively.

Each structure *Mtb*IMPDH2ΔCBS•inhibitor complex contains one protein chain per asymmetric unit with one molecule of IMP and one molecule of inhibitor bound. The active sites are ordered and the electron densities for ligands and protein are well defined (Figs 4 and 5). The overall structures of the inhibitor complexes are very similar, with r.m.s.d. for Cα atoms of A chains ranging from 0.23 to 0.46 Å. The structures of complexes with **MAD1** and **Q67** contain a potassium ion, as described for the apo protein. The crystallization buffer used to obtain the IMP•**P41** did not contain potassium ions (Table 4). The protein-IMP interactions are analogous to those described above for XMP, as well as for previously determined structure of IMP complexes [21,27,29,30], and will not be discussed further.

The inhibitors have modular structures consisting of two aromatic moieties connected by different linkers (1,2,3-triazole in **MAD1**, urea in **P41** and an amide in **Q67**) (Fig 1C). One aromatic portion of the inhibitor stacks against the hypoxanthine ring of IMP and interacts with residues within the nicotinamide-binding subsite (the “leftside” rings, Fig 1C). The other aromatic moiety interacts with IMSM residue Y487’ in the A^B^-subsite (the “rightside” rings; Fig 1C) and the linker bends around IMSM residue A285. Specific interactions for each inhibitor will be described below.

### *Mtb*IMPDH2ΔCBS•IMP•MAD1 complex

**MAD1** is a mycophenolic acid-adenosine conjugate initially designed to target human IMPDHs [16]. It was the first reported inhibitor of *Mtb*IMPDH2, although it is a much more potent inhibitor of the human enzymes. Other mycophenolic acid-adenosine conjugates with varying linkers did not inhibit *Mtb*IMPDH2. The mycophenolic acid-derived moiety interacts with IMP and forms hydrogen bonds with T343, G334 and G336 (Fig 4A). These residues are conserved in all IMPDHs and the corresponding interactions are also present in eukaryotic enzymes (for example, in the E-XMP*•mycophenolic acid complex of Chinese hamster IMPDH2 (PDB id 1JR1) [41]). **MAD1** does not interact with E458, in contrast to the *Cp*IMPDH inhibitors (see below). The absence of these contacts may account for the relatively low affinity of this compound (S9 Table). The triazole linker of **MAD1** engages in hydrogen bonding interaction via the N3 atom with the main chain amide nitrogen atom of A285, while the remainder of the triazole makes van der Waals contacts with R108 and T284 (Fig 4A). The position of the adenosine portion of **MAD1** in the A^B^-subsite is superimposable with the position of the adenosine moiety of NAD^+^ in the XMP•NAD^+^ complex. Consequently, interactions with H286, N289, V60’, P61’, A483’ and Y487’ are maintained (Fig 4B). Thus, **MAD1** adopts an orientation analogous to the folded cofactor conformation that is preferred in bacterial IMPDHs.

The divergence of the cofactor binding sites in bacterial and eukaryotic enzymes is illustrated by comparing the structures of the *Mtb*IMPDH2ΔCBS•IMP•**MAD1** with hIMPDH2 in complex with another **MAD** inhibitor, C2-MAD, and ribavirin 5’-monophosphate (RVP)(PDB id 1NF7; doi:10.2210/pdb1nf7/pdb [39]). C2-MAD is a second generation mycophenolic adenine analogue that has a methylenebis(phosphonate) linker. The linker and mycophenolic acid-derived portions of the inhibitors are positioned similarly in both enzymes. However, the adenosine portion of C2-MAD rotates away from the subunit interface and binds in the A^E^-subsite (Fig 4C). The C2-MAD adenine ring π/π stacks between the side chains of H253 and F282 (hIMPDH2 numbering) located within a single monomer (Fig 4C). Thus, C2-MAD assumes an orientation analogous to the extended NAD^+^ conformation preferred in eukaryotic IMPDHs (Fig 3E). Therefore, interaction of the adenine moiety with the A^E^-subsite may account for higher affinity of MAD inhibitors with eukaryotic IMPDHs.

### *Mtb*IMPDH2ΔCBS •IMP•P41 complex

**P41** has a urea linker connecting the 3-isoprenyl-α,α-dimethylbenzyl (left side ring) and 4-chloro-3-*N*,*N*-dimethylbenzamide [26] (right side ring; Fig 1C). The hypoxanthine ring of IMP interacts with the sp^2^ center and the aromatic centroid of the left side ring (Figs 1C and 5A). Both nitrogen atoms of the urea linker form hydrogen bonds with the side chain of E458 (N1/**P41—**OE2/E458 and N2/**P41—**OE2/E458 distances of 3.27 and 3.08 Å, respectively) (Fig 5A). The interactions with E458 are observed in other inhibitor complexes and are likely to be important for inhibitor potency [21,27,29,30]. Within the A^B^-subsite, the right side ring interacts with the ring of Y487’ in an orientation that is midway between face-to-face and edge-to-face geometries. The right side ring also makes contacts with H286, V60’, and P61’.

The importance of the 4-chloro substituent for antitubercular activity can be explained by the presence of a pair of orthogonal halogen/hydrogen bonds [47,48]. The Cl atom interacts with the carbonyl of G486’ (Cl/**P41** –O/G486’ distance of 3.52 Å) (Fig 5A). This carbonyl also makes a hydrogen bond with the side chain of S57’ (O/G486’–OG/S57’ distance of 2.85 Å) at an angle of 90° relative to the halogen bond. The halogen substituent also contacts the imidazole ring of H286. The structure explains the diversity of carboxamide substitutions in the **P** compounds with antitubercular activity. The carboxamide group of **P41** does not participate in any hydrogen bonds with the protein. The two methyl groups sit in a hydrophobic pocket formed by T284, V261, A285, N289, L291 and V292. These hydrophobic interactions can explain the higher affinity of substituted amides (Table 2, compare P34 and P67, P146 and P150).

### *Mtb*IMPDH2ΔCBS •IMP•Q67 complex

Compound **Q67** contains 2,3-dichloroaniline (left side ring) and 2-(4-pyridyl)-1,3-benzoxazole moieties (right side ring) connected with an amide linker [27] (Fig 1C). The leftside ring interacts with IMP via π/π stacking and C-X/π contacts involving the 2-chloro substituent. The 3-chloro substituent contacts the carbonyl atom of M424 and interacts with G425 (Fig 5B). These interactions explain the prevalence of the 2,3-dichloro substituents in the set of Q compounds with antitubercular activity. As in **P41**, the nitrogen atom of compound **Q67** amide linker hydrogen bonds with one of the side chain oxygen atoms of E458 but the interaction for **Q67** is stronger (2.79 Å versus 3.08 and 3.27 Å for **P41**).

As observed for the other inhibitors, the 4-pyridyl–1,3-benzoxazole moiety of **Q67** is held in place by interactions with H286, V60’, P61’, and Y487’ in the A^B^-site. In addition, **Q67** makes several unique contacts: the N3 atom of the 1,3-benzoxazole group interacts with the main chain nitrogen atom of H286 and two water molecules connect the N4 atom of the 4-pyridyl substituent with the main chain nitrogen atom of R290 and the carbonyl oxygen atom of S57’ (Fig 5B). Similar water-mediated interactions involving the pyridyl moiety were previously observed in the structures *Cp*IMPDH and *Ba*IMPDH complexes with another Q-series inhibitor, **Q21** [21,27]. The additional interactions of chloro substituents, the short hydrogen bond with E458, and the network of water-mediated contacts can account for the high potency of **Q67**. The structure also explains why the stereochemistry of the linker is essential. The (*S*)-methyl group faces away from E458 and interacts with L455 and M430. The methyl group in the (*R*) isomer is most likely oriented towards E458 and thus disrupts the crucial hydrogen bond interaction between the amide linker and E458, making this isomer inactive.

## Discussion

The structure of *Mtb*IMPDH2ΔCBS•XMP•NAD^+^ confirms our recent findings that bacterial IMPDHs bind NAD^+^ in an unusual compact conformation with the A^B^-subsite located at the interface between two monomers. The different locations of the A^B^ and A^E^ sites account for the selectivity of the *Cp*IMPDH inhibitors for bacterial IMPDHs. All high affinity *Cp*IMPDH inhibitors bind to bacterial enzymes using the A^B^ site. In contrast, it appears that MAD inhibitors bind with high affinity to human IMPDH using the A^E^ site and with low affinity to bacterial enzymes using A^B^ site. Interestingly, the nicotinamide ring in the *Mtb*IMPDH structure is in a *syn*-conformation, which is not compatible with the *pro-S* hydride transfer observed in the reaction. Similar *syn* NAD(H) conformations have been observed among other pro-*S* stereospecific enzymes, such as transhydrogenase [49] and UDP-galactose 4-epimerase [50]. We link the ability to bind the *syn*-conformation with the presence of the IMSM that is essential for inhibitor binding [10,30,31]. While hydride transfer cannot occur in the *syn*-conformation, it is possible that this conformation has another regulatory role.

New antibiotics to treat tuberculosis are urgently needed. Our work identifies inhibitors of *Mtb*IMPDH2 with encouraging antitubercular activity. We also report the first crystal structures of this promising antimicrobial drug target, including the apo form and complexes with XMP/NAD^+^ and three structurally distinct inhibitors. These structures will greatly facilitate the further development of *Mtb*IMPDH2-targeted antibiotics. Our *Mtb*IMPDH2ΔCBS inhibitor complexes provide important insights into the interactions that modulate affinity as well identify possible locations for further inhibitor optimization for both potency and cellular accumulation. Interactions with the hypoxanthine moiety of IMP, E458, and A^B^-subsite are especially important for high affinity and selectivity. It is important to note that while the active site flap is partially disordered in all *Mtb*IMPDH2ΔCBS structures, different conformations of the flap are observed in the apo structure and the structures of the complexes. Thus, the flap may transiently interact with the inhibitor and these interactions may also contribute to inhibitor affinity.

## Material and Methods

### Materials

IMP was purchased from Acros Organics. NAD^+^ and NADH were purchased from Roche and Sigma, respectively. Tris, and common chemicals were purchased from Sigma. KCl and trichloroacetic acid were purchased from Fisher. *Cp*IMPDH and *Ba*IMPDH were purified as previously described [27,32]. The synthesis of the *Cp*IMPDH inhibitors has been reported previously [23–25,27–30,37]. The synthesis of **MAD1** is described in [16]. Crystallization reagents were purchased from Hampton Research and Microlytic.

### Values of cLogP and tPSA

Values of clogP and tPSA were calculated in ChemBioDraw (Cambridgesoft Inc.). The relevant ionizations at pH 7.4 were included in the structures [37].

### Cloning

The CBS domain deletion mutant (*Mtb*IMPDH2ΔCBS) was constructed via the megaprimer cloning method [51]. Wild type *Mtb*IMPDH2 clone in vector pMCSG7 [27] was used as a template. The E126-R252 (ΔCBS) deletion primer and the *Mtb*IMPDH2 coding sequence forward primer were used to amplify a region of *Mtb*IMPDH2-pMCSG7 ranging from the residue M1 to residue V261 with 5’ LIC overhang, while replacing codons for residues E126-R252 with codons for GG. The resulting product was used as a megaprimer in the whole plasmid synthesis reaction, with *Mtb*IMPDH2-pMCSG7 as template and a reverse primer encoding 3’ end of *Mtb*IMPDH2. KOD Hot Start DNA polymerase kit (EMD Millipore) was utilized in a PCR reaction. Cycling was performed at 95° for 3 min, followed by 95° for 40 sec, 53° for 40 sec, 72° for 1.5 min for 32 cycles. The PCR product was treated with T4 polymerase (Promega), annealed into pMSCG7 vector, transformed into *E*. *coli* BL21(DE3) cells carrying the pMAGIC plasmid encoding rare *E*. *coli* tRNA (Arg (AGA/AGG)) [52] and the resulting clone sequenced.

### Stereochemistry of hydride transfer

[2-^2^H]-IMP or [2-^1^H]-IMP (2 mM) is mixed with NAD^+^ (2 mM) in assay buffer (50 mM d–11 Tris, 150 mM KCl, 1 μM DTT, pD 8.0) in D_2_O. *Mtb*IMPDH (1 μM) or hIMPDH2 (1.3 μM) was added to initiate the reaction. After 2h incubation at room temperature, the protein was separated from small molecules by centrifugation using Amicon centrifugal filter (Millipore, 10K cutoff), and the small molecule mixtures were directly analyzed by 400MHz ^1^H NMR with water suppression using presaturation pulse.

### MIC determinations

MIC is concentration that completely inhibits growth. Compounds MICs were determined as previously described [53]. MIC values were determined in at least triplicate according to the broth microdilution methods using compounds from DMSO stock solutions. Isoniazid was used as a positive control and DMSO was utilized as a negative control. Isolated *Mtb* cells (ATCC 27294) were cultured to an OD 0.2–0.3 in the required medium, then diluted to deliver approximately 1 x 10^4^ bacteria per well of a 96 well clear round-bottom plate. Plates were read after 1 week with an inverted enlarging mirror plate reader and graded as either growth or no growth. GAST/Fe medium (per liter) consisted of 0.3 g of Bacto Casitone (Difco), 4.0 g of dibasic potassium phosphate, 2.0 g of citric acid, 1.0 g of L-alanine, 1.2 g of magnesium chloride hexahydrate, 0.6 g of potassium sulfate, 2.0 g of ammonium chloride, 1.80 ml of 10 sodium hydroxide, and 10.0 ml of glycerol, 0.05% Tween 80 and 0.05 g of ferric ammonium citrate adjusted to pH 6.6. 7H9/glycerol/glucose/BSA/Tween medium consisted of Middlebrook 7H9 broth base supplemented per liter with 0.2% glucose, 0.2% glycerol, 0.5% BSA fraction V, 0.08% NaCl and 0.05% Tween 80.

Details of protein expression, purification, crystallization, data collection, X-ray structure solution and refinement, steady state kinetic measurements and inhibition experiments are listed in S1 Appendix.

## Supporting Information

S1 Appendix Details of protein expression, purification, crystallization, data collection, X-ray structure solution and refinement, steady state kinetic measurements and inhibition experiments. (DOCX)

S1 Fig Multiple sequence alignments of IMPDHs. (A) Sequence alignment of three *Mtb* IMPDHs: *Mtb*IMPDH1 (*guaB1*; gi: 15608980), *Mtb*IMPDH2 (*guaB2*; gi: 15610547) and *Mtb*IMPDH3 (*guaB3*; gi: 444896966). Secondary structure elements derived from *Mtb*IMPDH2ΔCBS (PDB code 4ZQR) are depicted as arrows (representing β-strands), coils (representing α- and 3_10_-helices), TT (strict β-turns) and TTT (strict α-turns). The location of CBS domain is shown as a green line. The position of catalytic Cys residue is indicated by a yellow rectangle. It is important to note that *Mtb*IMPDH3 (*guaB3*) does not posses the catalytic Cys residue suggesting that this protein may not be an IMPDH enzyme. (B) Sequence alignment of bacterial and human IMPDHs discussed in this study. The sequences include *Mtb*IMPDH1 (*guaB1*; gi: 15608980), *Mtb*IMPDH2 (*guaB2*; gi: 15610547), *B*. *anthracis* str. Ames (gi: 30253523), *V*. *cholera* O1 biovar (gi: 15640786), human type I (gi: 217035148), human type II (gi: 66933016) and *C*. *parvum* (gi: 323510309). *Mtb*IMPDH3 was omitted. Secondary structure elements derived from *Ba*IMPDH (PDB code 3TSB) are depicted as in panel A. The location of CBS domain is shown as a *green line*. The position of catalytic Cys residue is indicated by a yellow rectangle. Positions of residues involved in binding of the NAD^+^ adenosine moiety in bacterial (A^B^-subsite) and eukaryotic (A^E^-subsite) enzymes are indicated by purple and black rectangles, respectively. In both panels identical residues are highlighted in red, and similar residues are shown as red letters. The alignment was generated using MultiAlin [54] and ESPript [55] programs.
(TIFF)

S2 Fig Comparison of the effect of inhibitors on *Mtb*IMPDH and *Ba*IMPDH. The line denotes equal values of *K*_i,app_.
(TIFF)

S3 Fig Comparison of MIC for antitubercular activity and *K*_i,app_ for inhibition of *Mtb*IMPDH. (TIFF)

S4 Fig *Mtb*IMPDH2ΔCBS catalyzes the transfer of hydride to the *pro-S* face of NAD^+^. NMR spectra of IMPDH reaction mixtures after 2 h incubation of NAD^+^ with (A) hIMPDH2 (1.3 μM) and IMP; (B) hIMPDH2 (1.3 μM) and [2-^2^H]-IMP; (C) *Mtb*IMPDH2ΔCBS (1 μM) and IMP; (D) *Mtb*IMPDH2ΔCBS (1 μM) and [2-^2^H]-IMP. The peaks assigned to protons on C–4 of NADH nicotinamide ring are shown.
(TIFF)

S1 Table Structures of inactive A series amide derivatives. All values are the average of at least two determinations unless otherwise noted. a. Data from [24]. b. Data from [23]. c. Data from [37]. d. Single determination.
(DOCX)

S2 Table Structures of inactive A series triazole derivatives. a. Data from [24]. b. Data from [37]. c. Single determination.
(DOCX)

S3 Table SAR of enzyme inhibition for C series benzimidazole derivatives. n.d. = not determined. a. *Cp*IMPDH data from [30]. b. Data from [25]. c. *Ba*IMPDH data from [37]. c. Single determination.
(DOCX)

S4 Table Structures of inactive D series phthalazinone derivatives. n.d. = not determined. a. Data from [28]. b. Data from [37]. c. Single determination.
(DOCX)

S5 Table Structures of inactive P compounds. n.d. = not determined. a. *D*ata from [26]. b. *D*ata from [37].
(DOCX)

S6 Table P series: SAR of enzyme inhibition for the isopropyl and urea group. n.d. = not determined. a. Data from [26]. b. Data from [37].
(DOCX)

S7 Table Structures of inactive Q compounds. n.d. = not determined. n.d. = not determined a. Data from [27]. b. Data from [37].
(DOCX)

S8 Table *Cp*IMPDH inhibitors with antitubercular activity: comparison of enzyme inhibition. n.d. = not determined. a. Data from [26]. b. Data from [27]. c. Data from [37]. d. Single determination. e. Two determinations.
(DOCX)

S9 Table Inhibition of *Mtb*IMPDH2ΔCBS. * Indicates tight binding conditions. C, competitive inhibition; UC, uncompetitive inhibition, NC, noncompetitive inhibition.
(DOCX)
