# Supplementary material for: Mycobacterium tuberculosis IMPDH in Complexes with Substrates, Products and Antitubercular Compounds
Source: PLoS One. 2015 Oct 6;10(10):e0138976. doi: 10.1371/journal.pone.0138976 (PMC4594927; doi:10.1371/journal.pone.0138976)
Supplement: S1 Appendix — (DOCX) [file pone.0138976.s001.docx]

**Appendix S1 for**

***Mycobacterium tuberculosis* IMPDH in complexes with substrates, products and antitubercular compounds**

Magdalena Makowska-Grzyska, Youngchang Kim, Suresh Kumar Gorla, Yang Wei, Kavitha Mandapati, Minjia Zhang, Natalia Maltseva, Gyan Modi, Helena I. Boshoff, Minyi Gu, Courtney Aldrich, Gregory D. Cuny, Lizbeth Hedstrom* and Andrzej Joachimiak*

**Protein expression, purification and crystallization**

*Mtb*IMPDH2ΔCBS protein was expressed and purified for functional and crystallographic studies following a previously described procedure [[1](#_ENREF_1),[2](#_ENREF_2)]. The protein was appended to an N-terminal His_6_-tag and purified using nickel(II) affinity chromatography (IMAC). The His-tag was subsequently removed with TEV protease and the His-tag-free protein was additionally purified using a subtractive IMAC to remove the released tag and uncut protein.

Crystallization screening was set-up with the help of a Mosquito liquid dispenser (TTP LabTech) using the sitting-drop, vapor-diffusion method in 96-well CrystalQuick plates (Greiner Bio-One). For co-crystallization trials, ligands were used at a 4–10 fold molar excess over protein concentration. For each condition, 0.4 μl protein solution and 0.4 μl crystallization formulation were mixed and the mixture was equilibrated against a 135 μl reservoir. INDEX, SaltRx, and four MCSG crystallization screens were used. Diffraction quality crystals typically appeared within 2–7 days. Crystals of *Mtb*IMPDH2ΔCBS•XMP•NAD^+^ were obtained by soaking crystals containing the *Mtb*IMPDH2ΔCBS•IMP complex with a 200 mM aqueous NAD^+^ solution for 12 hours at 16°C, followed by cryo-protection. Crystallization conditions are listed in Table 4.

**Data collection**

Prior to flash-cooling in liquid nitrogen, all crystals were cryoprotected in an appropriate cryoprotectant solution. The crystals were mounted on Litholoops (Molecular Dimensions, Apopka, FL). All X-ray diffraction experiments were performed at the Structural Biology Center 19-ID beamline at the Advanced Photon Source, Argonne National Laboratory [[3](#_ENREF_3)]. The HKL3000 suite [[4](#_ENREF_4)] was used to process, merge, and scale. The processing and scaling statistics are given in Table 4.

**Structure solution and refinement**

All diffraction data were collected at 100 K. The single wavelength data at 0.97915 Å up to 1.69 Å were collected from a single crystal of *Mt*IMPDH2ΔCBS. The crystal was exposed for 3 s per degree rotation of ω for total 220º with the crystal to detector distance of 230 mm. The single wavelength (0.97899 Å) data for the crystals of the inhibitor complexes were collected using similar protocols. For *Mt*IMPDH2ΔCBS•XMP•NAD^+^ complex, the data were collected to 1.60 Å, 3 s exposure/deg for 144º with the distance of 230 mm 0.9792 Å. For **MAD1**, the data were collected to 1.89 Å, 3 s exposure/deg for 110º with the distance of 310 mm; for **P41**, to 2.0 Å, 3 s exposure/deg for 120º with the distance of 300 mm, and finally for **Q67**, to 1.76 Å, 3 s exposure/deg for 110º with the distance of 260 mm. All data were recorded on a CCD detector ADSC Q315r. The SBC-Collect program was used for all data collection. Data collection strategy, integration, and scaling were performed with the HKL3000 program package [[4](#_ENREF_4)]. Summary of the crystallographic data can be found in Table 4.

The structure of the apo form of *Mt*IMPDH2ΔCBS was determined by molecular replacement with HKL3000 (Molrep/refmac) using chain A of the ΔCBS mutant of *Clostridium perfringens* IMPDH (PDB ID 4Q32; 2.79 Å) [[2](#_ENREF_2)] as a search model after any ligands and water molecules were removed. Rigid-body refinement was done at 3.0 Å and the initial refinement was done at 1.70 Å as part of HKL3000 molecular replacement procedure [[4](#_ENREF_4)]. The initial model contained 4 copies of the search model. Extensive manual model building with coot [[5](#_ENREF_5)] and the subsequent refinement using phenix.refine [[6](#_ENREF_6)] was performed against the full data set up to 1.70 Å until the structure converged to the *R* factor (*R_work_*) of 0.155, and *R_free_* of 0.182 with the r.m.s.d. for bond distances of 0.010 and the r.m.s.d. for bond angles of 1.277°. The asymmetric unit of the triclinic space group *P*1 was composed of four protein chains, A, B, C and D as a functional tetrameric unit. The protein construct *Mt*IMPDH2ΔCBS used in this study contained residues 1-125 and 253-528 with GG linker inserted in place of the CBS domain (E126-R252). The numbering of residues in the protein residues followed the original protein sequence to avoid the confusion. The GG linker was well ordered and visible in all chains. A number of C-terminal as well as several N- terminal residues that had been introduced as a cloning artifact (SNA) [[7](#_ENREF_7)] were missing due to disorder. Chain A was comprised of residues 27-528, chain B included residues 27-528, chain C contained residues 26-528 and chain D was comprised of residues 27-528. In addition, several residues within the active site flap were disordered and were not modeled. These included residues 432-451 in all four chains. The final model also contained four potassium ions (one per chain), four phosphate molecules, 799 ordered water molecules and 23 other small molecules such as 1,2-propanediol and glycerol that were used in the purification and crystallization.

The structures of *Mt*IMPDH2ΔCBS complexes with XMP and NAD^+^ and inhibitors were determined by molecular replacement using chain A of the structure of the apo-form of *Mt*IMPDHΔCBS. In each complex, the presence of XMP/NAD^+^ or IMP and an inhibitor in the active site was apparent from the initial electron density map (*F_o_*) calculated without any ligand molecule. Extensive manual model building with coot [[5](#_ENREF_5)] and the subsequent refinement using phenix.refine [[6](#_ENREF_6)] was performed against the full data set up to the full resolution until each structure converged. The final R_work_ and R_free_ for the *Mt*IMPDH2ΔCBS•XMP•NAD^+^ complex were 0.160 and 0.191, respectively, and r.m.s.d. for bonds and angles were 0.009 and 1.325, respectively and for **MAD1,** R_work_ and R_free_ were 0.148 and 0.189, respectively, and r.m.s.d. for bonds and angles were 0.006 and 1.192, respectively. For **P41**, R_work_ was 0.174 and R_free_ was 0.222, and r.m.s.d. for bonds and angles were 0.012 and 1.507, respectively. Finally, for **Q67**, R_work_ and R_free_ were 0.153 and 0.179, and r.m.s.d. for bonds and angles were 0.007 and 1.160, respectively. The detailed refinement statistics for all structures are shown in Table 4.

All IMPDH-inhibitor complexes and the XMP/NAD^+^ complex were crystallized into the body-centered tetragonal space group *I*4 and the asymmetric unit contained only one chain of a 4-fold symmetric tetramer. Several N- and C-terminal residues were missing due to disorder in all inhibitor complexes. The *Mt*IMPDH2ΔCBS•XMP•NAD^+^ complex contained residues 28-509. Complex with **MAD1** was comprised of residues 28-528, complex with **P41** included residues 28-525, and the complex with **Q67** contained residues 28-527. In all structures several residues within the active site flap were disordered and were not modeled. These included residues 431-454 for the XMP/NAD^+^ complex, 432-453 for **MAD1**, 432-454 for **P41**, and 433-453 for **Q67**. The GG linker that replaced the CBS domain (residues 126-252) was well visible in all inhibitor complexes. The final models also included one IMP molecule and one molecule of the appropriate inhibitor (one XMP and one NAD^+^ molecule for the XMP/NAD^+^ complex), as well as solvent molecules. Thus, there were 161 ordered water molecules for the XMP/NAD^+^ complex, 112 water molecules for **P41**, while complexes with **MAD1** and **Q67** contained 169 and 188 water molecules, respectively. Structures of **P41** and **Q67** also included one potassium ion, one glycerol molecule and one 1,2-propanediol molecule that were most likely derived from purification and/or crystallization buffers.

The stereochemistry of the structure was checked with PROCHECK [[8](#_ENREF_8)] and the Ramachandran plot. Atomic coordinates and experimental structure factors of the structures have been deposited in the PDB under the ID codes, 4ZQM for *Mt*IMPDH2ΔCBS•XMP•NAD^+^, 4ZQP for **MAD1**, 4ZQN for **P41**, and 4ZQO for **Q67**, and 4ZQR for the apo structure.

**Steady state kinetics**

The steady state kinetics parameters of *Mtb*IMPDH2ΔCBS were obtained by measuring initial velocities at varying concentrations of IMP and NAD^+^ by monitoring the production of NADH in absorbance at 340 nm (ε = 6.22 mM^-1^•cm^-1^) using Hitachi U-2000 or Cary 100 Bio spectrophotometer. All the measurements were done in the assay buffer (50 mM Tris, 150 mM KCl, 1 mM DTT, pH 8.0) at 25°C with 125 nM enzyme in a total of 1 ml volume in 1 cm pathlength cuvettes. The value of *K_m•IMP_* was obtained by fitting the initial velocities measured at the fixed concentration of NAD^+^ (3 mM) and varying concentrations of IMP (2 to 1500 μM) into the Michaelis-Menton equation (1):

*v* = *V_max_*•[S] / (*K_m_*+[S]) (1)

Similarly, the values of *K_m•NAD_* and *K_ii•NAD_* were determined by measuring the initial velocities at fixed concentration of IMP (1 mM) and varying concentrations of NAD^+^ (0.1 to 10 mM) and fitting into the uncompetitive substrate inhibition equation (2):

*v* = *V_max_* / (1 + *K_m_*/[S] + [S]*/K_ii_*) (2)

where *v* is the initial velocity, *V_max_* is the maximal velocity, [S] is the substrate concentration, *K_m_* is the Michaelis constant, and *K_ii_* is the intercept inhibition constant. The k_cat_ value is the average of both conditions. All the data fitting was performed with SigmaPlot program.

**Enzyme inhibition**

The *K_i,app_* values were determined by measuring the initial velocities at varying concentrations of the inhibitors (**P41**, 1-1000 nM; **Q67**, 1-10,000 nM; **MAD1**, 0.01-100,000 nM) with fixed concentrations of IMP (0.5 mM) and NAD^+^ (1.5 mM). The values of *K_i,app_* were obtained using the equations (3) and (4)

*v_i_* = *v_0_* / (1+ [I]/ IC_50_) (3)

K_i,app_ = IC_50_ – [E]/2 (4)

where *v_i_* is the initial velocity with inhibitor *I* present at the concentration [I], and *v_0_* is the initial velocity in the absence of the inhibitor. If the IC_50_ value is comparable to the enzyme concentration, the Morrison tight binding equation was used to determine *K_i,app_* (5)

*v_i_*/*v_0_* = 1-(([E] + [I] +K_i,app_) - (([E] + [I] + K_i,app_)^2^ - 4[E][I])^0.5^)/(2[E]) (5)

where [E] is the concentration of the enzyme. All the initial velocity measurements were performed in triplicates. The *K_i,app_* values reported are the average of three independent experiments unless otherwise noted.

The mechanism of inhibition was determined by varying inhibitor and substrate concentrations at fixed concentrations of the other substrate. Data were fit to equations describing competitive, uncompetitive and noncompetitive inhibition mechanisms (Equation 6-8) using SigmaPlot program (SPSS, Inc.):

Competitive inhibition: v = *V_m_*[S]/{*K_m_*(1 + [I]/*K_is_*) + [S]} (6)

Uncompetitive inhibition: v = *V_m_*[S]/{*K_m_* + [S](1 + [I]/*K_ii_*)} (7)

Noncompetitive inhibition: v = *V_m_*[S]/{*K_m_*(1 + [I]/*K_is_*) + [S](1 + [I]/*K_ii_*)} (8)

where *K_ii_* and *K_is_* represent the intercept and slope inhibition constants, respectively. The best fits were determined by the relative fit error. The Morrison equation (5) was used to evaluate tight-binding inhibitors. The mechanism of inhibition was evaluated from the substrate dependence of K_i,app_ (Equations 8-10):

Competitive inhibition: *K_i,app_* = *K_is_*(1 + [S]*K_m_*) (9)

Uncompetitive inhibition: *K_i,app_* = *K_ii_*(1 + [*K_m_*/S]) (10)

Noncompetitive inhibition: *K_i,app_* = ([S] + *K_m_*) / {(*K_m_/K_is_*) + ([S]/*K_ii_*)} (11)

**Synthesis of inhibitor Q77**

Scheme S1 outlines the synthesis of **Q77.** Unless otherwise noted, all reagents and solvents were purchased from commercial sources and used without further purification. All reactions were performed under a nitrogen atmosphere in dried glassware unless otherwise noted. All NMR spectra were obtained using a 400 MHz spectrometer and conducted in CDCl_3_. For ^1^H NMR reported in  units ppm and are reference to tetramethylsilane (TMS). All chemical shift values are also reported with multiplicity, coupling constants and proton count. For ^13^C NMR reported in  units ppm and are reference to the peak at 39.51 ppm. Coupling constants (*J* values) are reported in hertz. Column chromatography was carried out on SILICYCLE SiliaFlash silica gel F60 (40-63 μm, mesh 230-400). High-resolution mass spectra (HRMS) were obtained using a Q-tof UE521 mass spectrometer (University of Illinois, SCS, and Mass Spectrometry Lab). Enantiomeric purity was determined using HPLC analysis on a Agilent 1100 series instrument equipped with a quaternary pump using a Chiralpak OD-H column (250 mm × 4.6 mm) at 25°C. The UV absorption was monitored at 220 nm, and the injection volume was 20 μL. HPLC gradient was 80% n-hexane and 20% isopropanol, and a flow rate of 1.0 mL/min was used.

**(S)-2-(2-chlorophenoxy)-N-(2-(pyridin-4-yl)benzo[d]oxazol-6-yl)propanamide**

^1^H NMR (DMSO-*d_6_*, 400 MHz) δ 1.62 (d, *J* = 6.0 Hz, 3H), 4.98 (q, *J* = 6.8 Hz, 1H), 6.99 (t, *J* = 7.6 Hz, 1H), 7.08 (d, *J* = 8.8 Hz, 1H), 7.29 (t, *J* = 8 Hz, 1H), 7.47 (d, *J* = 8.4 Hz, 1H), 7.66 (dd, *J*_1_ = 8.8 Hz, 1H), 7.82 (d, *J* = 9.2 Hz, 1H), 8.09 (d, *J* = 6 Hz, 2H), 8.23 (s, 1H), 8.84 (d, *J* = 6 Hz, 2H), 10.42 (s, 1H); ^13^C NMR (DMSO-*d_6_*, 100 MHz) δ 18.5, 74.9, 110.7, 111.1, 115.0, 119.1, 120.7, 122.1, 122.3, 128.2, 130.2, 133.3, 135.9, 141.3, 146.7, 150.8, 152.8, 160.9, 169.3; ESI-HRMS for C_21_H_17_N_3_O_3_Cl (M+H)^+^ calcd. 394.0958 found 394.0953. Chiral purity (% ee > 98, t_R_ = 24.2 min for major enantiomer and t_R_ = 19.6 min for the minor enantiomer).

**Supplementary Scheme S1. Synthesis of Q77.**

**References**

1. Kim Y, Babnigg G, Jedrzejczak R, Eschenfeldt WH, Li H, et al. (2011) High-throughput protein purification and quality assessment for crystallization. Methods 55: 12-28.

2. Makowska-Grzyska M, Kim Y, Maltseva N, Osipiuk J, Gu M, et al. (2015) A novel cofactor binding mode in bacterial IMP dehydrogenases explains inhibitor selectivity. J Biol Chem 290: 5893-5911.

3. Rosenbaum G, Alkire RW, Evans G, Rotella FJ, Lazarski K, et al. (2006) The Structural Biology Center 19ID undulator beamline: facility specifications and protein crystallographic results. J Synchrotron Radiat 13: 30-45.

4. Minor W, Cymborowski M, Otwinowski Z, Chruszcz M (2006) HKL-3000: the integration of data reduction and structure solution--from diffraction images to an initial model in minutes. Acta Crystallogr D 62: 859-866.

5. Emsley P, Cowtan K (2004) Coot: model-building tools for molecular graphics. Acta Crystallogr D 60: 2126-2132.

6. Adams PD, Afonine RV, Bunkoczi G, Chen VB, Echols N, et al. (2010) PHENIX: a comprehensive Python-based system for macromolecular structure solution. Acta Crystallogr D 66: 213-221.

7. Kim Y, Dementieva I, Zhou M, Wu R, Lezondra L, et al. (2004) Automation of protein purification for structural genomics. J Struct Funct Genomics 5: 111-118.

8. Laskowski RA, MacArthur MW, Moss DS, Thornton JM (1993) PROCHECK: a program to check the stereochemical quality of protein structures. J Appl Crystal 26: 283-291.

9. Maurya SK, Gollapalli DR, Kirubakaran S, Zhang M, Johnson CR, et al. (2009) Triazole Inhibitors of *Cryptosporidium parvum* Inosine 5'-Monophosphate Dehydrogenase. J Med Chem 52: 4623-4630.

10. Sharling L, Liu X, Gollapalli DR, Maurya SK, Hedstrom L, et al. (2010) A Screening Pipeline for Antiparasitic Agents Targeting *Cryptosporidium* Inosine Monophosphate Dehydrogenase. PLoS Negl Trop Dis 4: e794 (791-712).

11. Mandapati K, Gorla SK, House AL, McKenney ES, Rao SN, et al. (2014) Repurposing *Cryptosporidium* inosine 5’-monophosphate dehydrogenase inhibitors as potential antibacterial agents. ACS Med Chem Lett 5: 846-850.

12. MacPherson IS, Kirubakaran S, Gorla SK, Riera TV, D'Aquino JA, et al. (2010) The Structural Basis of *Cryptosporidium*-Specific IMP Dehydrogenase Inhibitor Selectivity. J Am Chem Soc 132: 1230-1231.

13. Kirubakaran S, Gorla SK, Sharling L, Zhang M, Liu X, et al. (2012) Structure-activity relationship study of selective benzimidazole-based inhibitors of *Cryptosporidium parvum* IMPDH. Bioorg Med Chem Lett 22: 1985-1988.

14. Johnson CR, Gorla SK, Kavitha M, Zhang M, Liu X, et al. (2013) Phthalazinone inhibitors of inosine-5'-monophosphate dehydrogenase from *Cryptosporidium parvum*. Bioorg Med Chem Lett 23: 1004-1007.

15. Gorla SK, Kavitha M, Zhang M, Liu X, Sharling L, et al. (2012) Selective and Potent Urea Inhibitors of *Cryptosporidium parvum* Inosine 5'-Monophosphate Dehydrogenase. J Med Chem 55: 7759-7771.

16. Gorla SK, Kavitha M, Zhang M, Chin JEW, Liu X, et al. (2013) Optimization of Benzoxazole-Based Inhibitors of *Cryptosporidium parvum* Inosine 5'-Monophosphate Dehydrogenase. J Med Chem 56: 4028-4043.
