# Supplementary figures and images for: Mycobacterium tuberculosis IMPDH in Complexes with Substrates, Products and Antitubercular Compounds
Source: PLoS One. 2015 Oct 6;10(10):e0138976. doi: 10.1371/journal.pone.0138976 (PMC4594927; doi:10.1371/journal.pone.0138976)

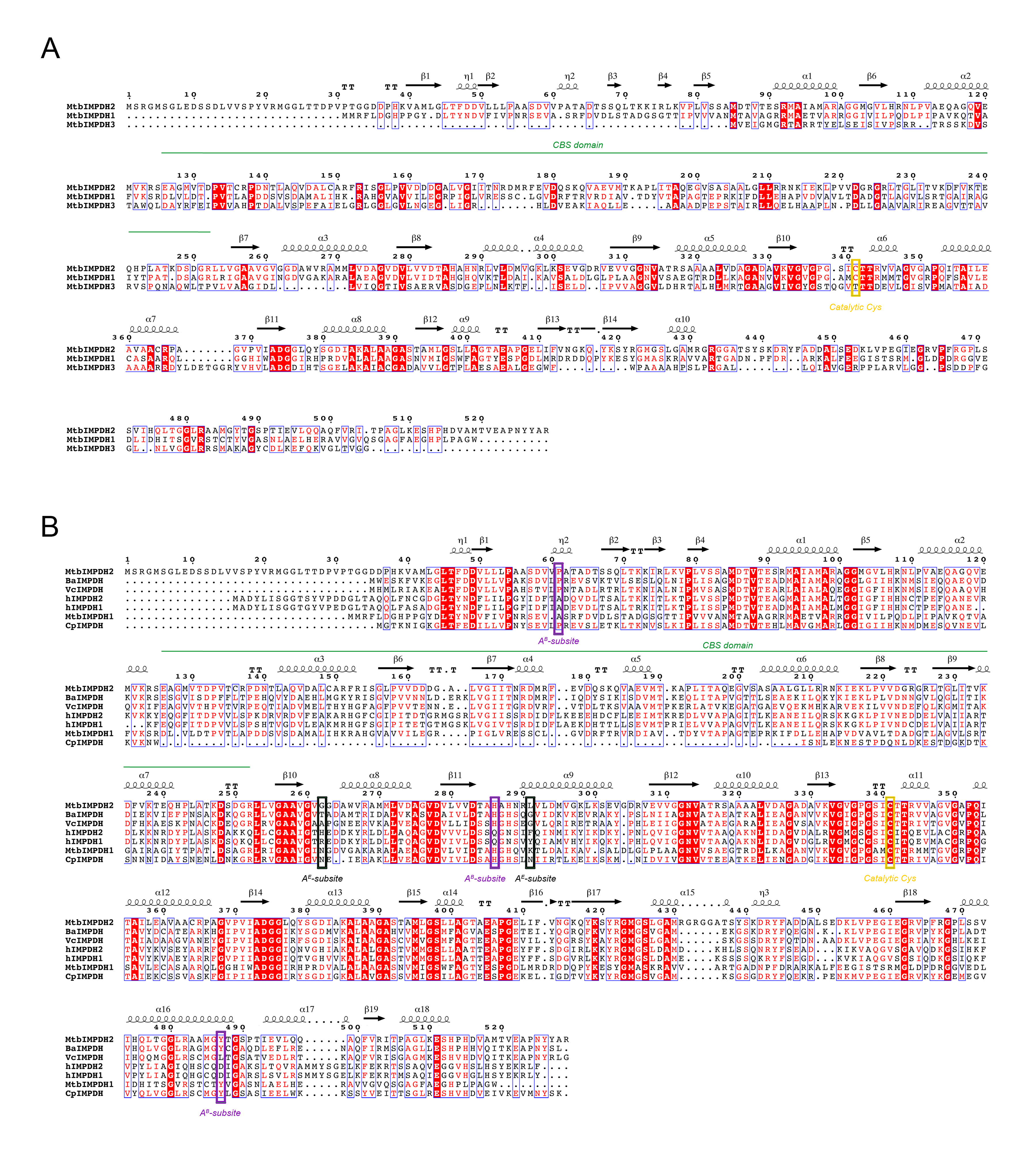

Supplement: S1 Fig — (A) Sequence alignment of three Mtb IMPDHs: MtbIMPDH1 (guaB1; gi: 15608980), MtbIMPDH2 (guaB2; gi: 15610547) and MtbIMPDH3 (guaB3; gi: 444896966). Secondary structure elements derived from MtbIMPDH2ΔCBS (PDB code 4ZQR) are depicted as arrows (representing β-strands), coils (representing α- and 310-helices), TT (strict β-turns) and TTT (strict α-turns). The location of CBS domain is shown as a green line. The position of catalytic Cys residue is indicated by a yellow rectangle. It is important to note that MtbIMPDH3 (guaB3) does not posses the catalytic Cys residue suggesting that this protein may not be an IMPDH enzyme. (B) Sequence alignment of bacterial and human IMPDHs discussed in this study. The sequences include MtbIMPDH1 (guaB1; gi: 15608980), MtbIMPDH2 (guaB2; gi: 15610547), B. anthracis str. Ames (gi: 30253523), V. cholera O1 biovar (gi: 15640786), human type I (gi: 217035148), human type II (gi: 66933016) and C. parvum (gi: 323510309). MtbIMPDH3 was omitted. Secondary structure elements derived from BaIMPDH (PDB code 3TSB) are depicted as in panel A. The location of CBS domain is shown as a green line. The position of catalytic Cys residue is indicated by a yellow rectangle. Positions of residues involved in binding of the NAD+ adenosine moiety in bacterial (AB-subsite) and eukaryotic (AE-subsite) enzymes are indicated by purple and black rectangles, respectively. In both panels identical residues are highlighted in red, and similar residues are shown as red letters. The alignment was generated using MultiAlin [54] and ESPript [55] programs. (TIFF) [file pone.0138976.s002.tiff]

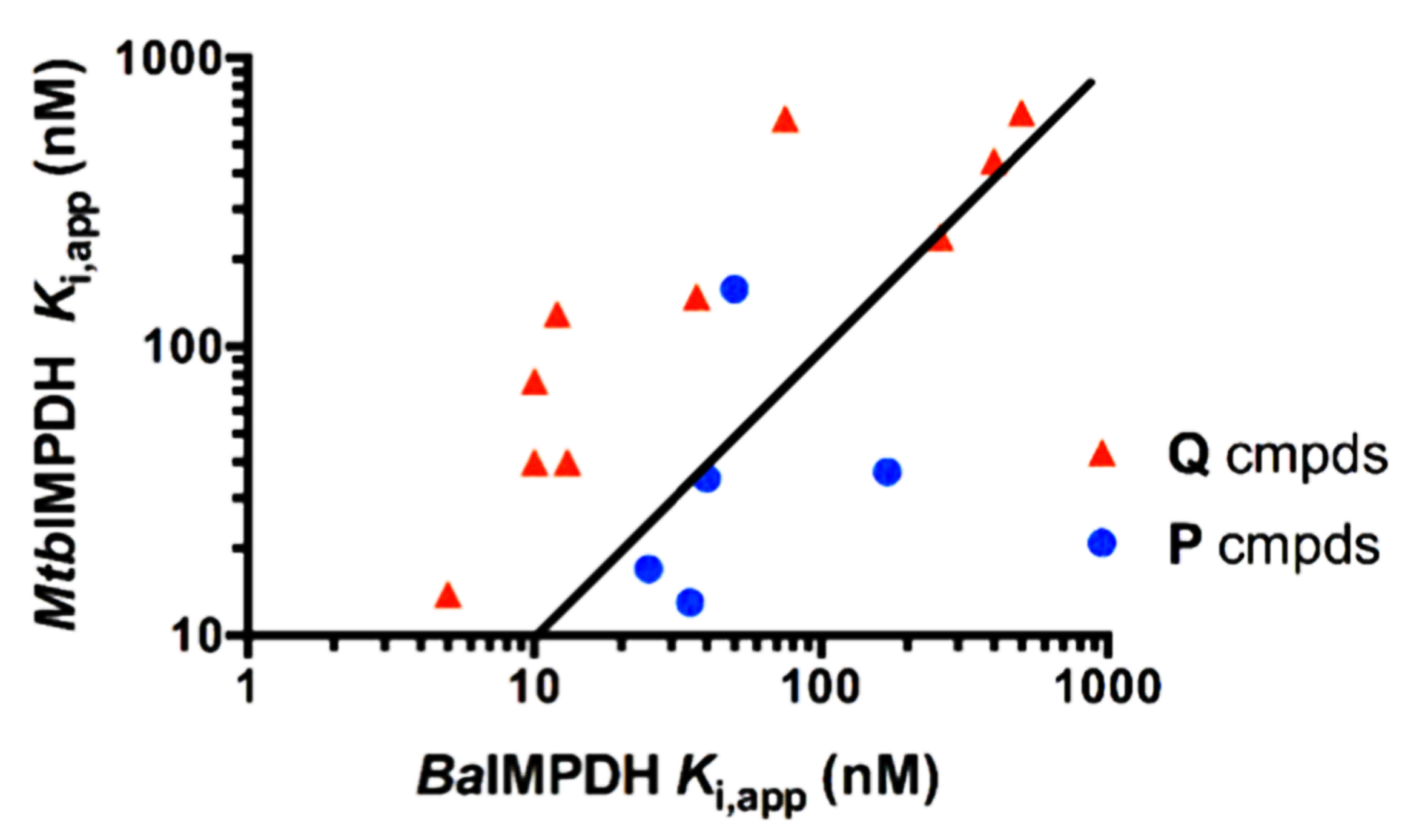

Supplement: S2 Fig — The line denotes equal values of K i,app. (TIFF) [file pone.0138976.s003.tiff]

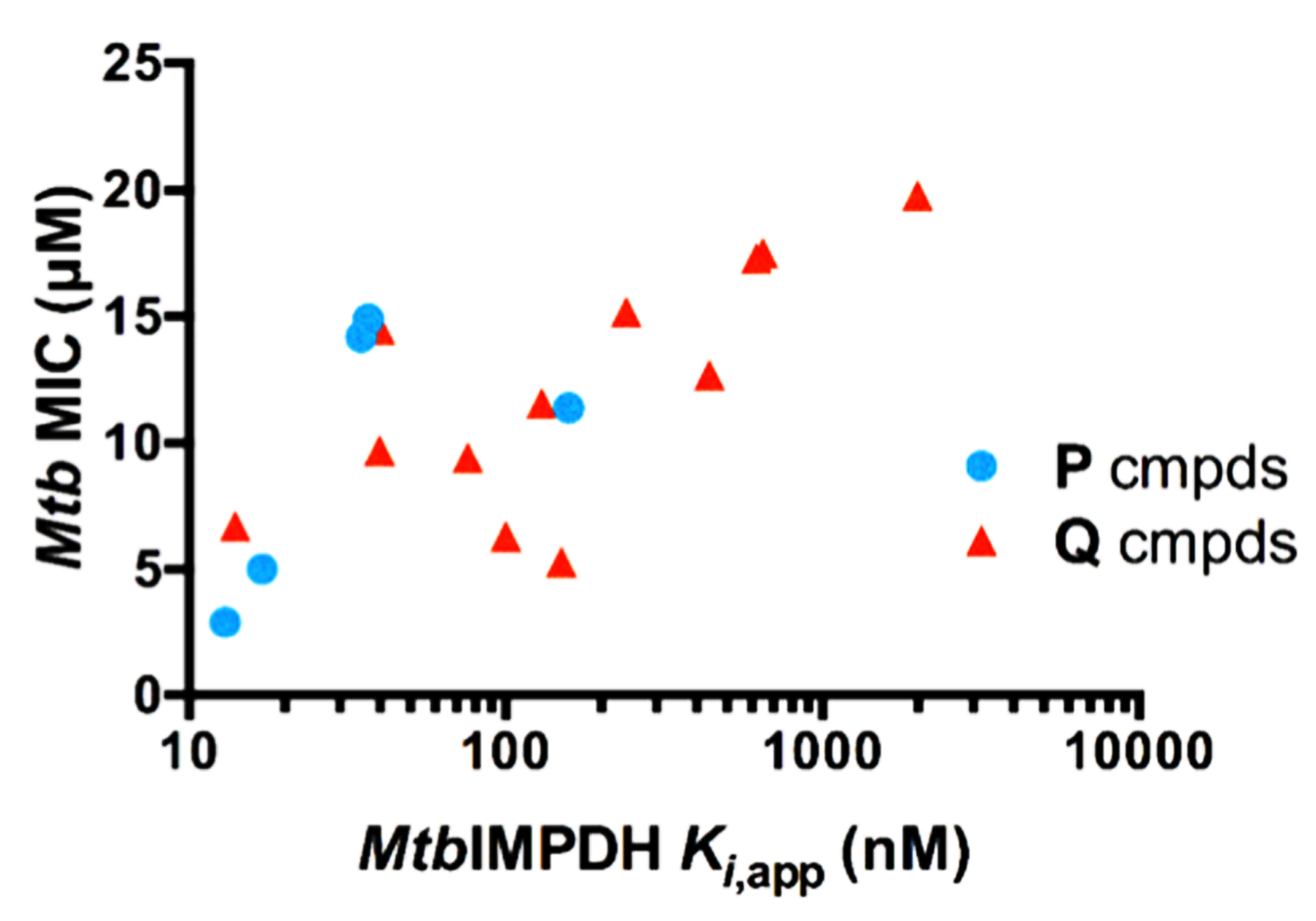

Supplement: S3 Fig — (TIFF) [file pone.0138976.s004.tiff]

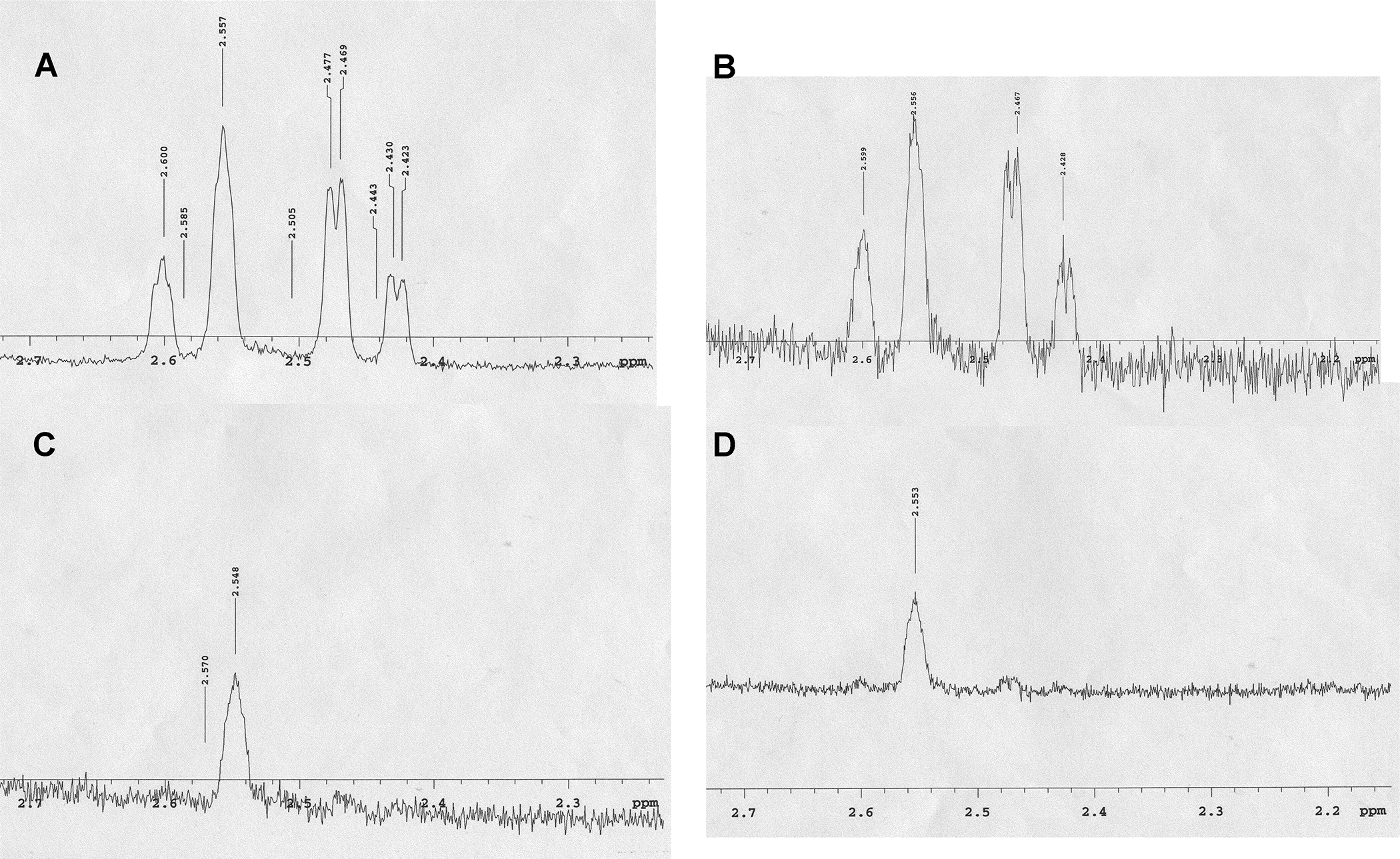

Supplement: S4 Fig — NMR spectra of IMPDH reaction mixtures after 2 h incubation of NAD+ with (A) hIMPDH2 (1.3 μM) and IMP; (B) hIMPDH2 (1.3 μM) and [2-2H]-IMP; (C) MtbIMPDH2ΔCBS (1 μM) and IMP; (D) MtbIMPDH2ΔCBS (1 μM) and [2-2H]-IMP. The peaks assigned to protons on C–4 of NADH nicotinamide ring are shown. (TIFF) [file pone.0138976.s005.tiff]
