# Supplementary material for: Mycobacterium tuberculosis IMPDH in Complexes with Substrates, Products and Antitubercular Compounds
Source: PLoS One. 2015 Oct 6;10(10):e0138976. doi: 10.1371/journal.pone.0138976 (PMC4594927; doi:10.1371/journal.pone.0138976)
Supplement: S1 Table — All values are the average of at least two determinations unless otherwise noted. a. Data from [24]. b. Data from [23]. c. Data from [37]. d. Single determination. (DOCX) [file pone.0138976.s006.docx]

**S1 Table. Structures of inactive A series amide derivatives.** All values are the average of at least two determinations unless otherwise noted. a. Data from [24]. b. Data from [24]. c. Data from [37]. d. Single determination.

| **** | | | | | |
| --- | --- | --- | --- | --- | --- |
| **Cmpd** | **R_1_** | **X** | **R_2_** | ***K_i,app_* (nM)** | |
|  |  |  |  | ***Cp*IMPDH** ^a^ | ***Ba*IMPDH** ^c^ |
| **A50** | Me | CH | 4-Cl | 1000 ± 100 ^a^ | 150 ± 30 ^c^ |
| **A61** | Me | CH | 4-Br | 161 ± 86 ^b^ | 165 ± 75 ^c^ |
| **A64** | Me | CH | 4-CF3 | 1500 ± 700 ^b^ | 650 ± 180 |
| **A67** | Me | N | 4-Cl | 660 ± 200 ^a^ | 1000 ± 80 ^c^ |
| **A68** | *i*-Pr | CH | 4-Br | 400 ± 200 ^b^ | 138 ^d^ |
| **A72** | *c*-Pr | CH | 4-Cl | > 5000 ^a^ | 160 ± 55 ^c^ |
