# Supplementary material for: Mycobacterium tuberculosis IMPDH in Complexes with Substrates, Products and Antitubercular Compounds
Source: PLoS One. 2015 Oct 6;10(10):e0138976. doi: 10.1371/journal.pone.0138976 (PMC4594927; doi:10.1371/journal.pone.0138976)
Supplement: S2 Table — a. Data from [24]. b. Data from [37]. c. Single determination. (DOCX) [file pone.0138976.s007.docx]

**S2 Table. Structures of inactive A series triazole derivatives.** a. Data from [24]. b. Data from [37]. c. Single determination.

|  | | | | | | |
| --- | --- | --- | --- | --- | --- | --- |
| **Cmpd** | **R_1_** | **R_2_** | **X** | **Y** | ***K_i,app_* (nM)** | |
|  |  |  |  |  | ***Cp*IMPDH** | ***Ba*IMPDH** |
| **A74** | Me | 4-Cl | CH | CH | 130 ± 30 ^a^ | 260 ± 70 ^b^ |
| **A90** | Me | 3,4-di-Cl | N | CH | 20 ± 10 ^a^ | n.d. |
| **A91** | Me | 4-CN | N | CH | 140 ± 30 ^a^ | 450 ^c^ |
| **A92** | Me | 3-Cl, 4-CN | N | CH | 40 ± 2 ^a^ | 400 ^c^ |
| **A93** | H | 4-Cl | N | CH | 400 ± 200 ^a^ | 800 ± 300 ^b^ |
| **A94** | H | 3,4-diCl | N | CH | 20 ± 10 ^a^ | 80 ± 30 ^b^ |
| **A98** | (*R*)-Me | 4-Cl | CH | N | 9 ± 1 ^a^ | 15 ± 4 ^b^ |
| **A100** | Me | 4-Cl | N^+^-O^-^ | CH | 30 ± 10 ^a^ | 60 ± 10 ^b^ |
| **A102** | Me | 4-Cl | CH | N^+^-O^-^ | 44 ± 2 ^a^ | 90 ± 30 ^b^ |
| **A103** | Me | 3,4-di-Cl | N^+^-O^-^ | CH | 18 ± 3 ^a^ | 60 ± 20 ^b^ |
| **A105** | (*R*)-Me | 3,4-di-Cl | N | CH | 9 ± 6 ^a^ | 20 ± 2 ^b^ |
| **A106** | (*R*)-Me | 3-Cl, 4-CN | N | CH | 31 ± 9 ^a^ | 340 ± 40 ^b^ |
| **A108** | (*S*)-Me | 3-Cl, 4-CN | N | CH | 60 ± 5 ^a^ | 3700 ± 60 ^b^ |
| **A109** | (*R*)-Me | 4-Cl | CH | N^+^-O^-^ | 24 ± 5 ^a^ | 40 ± 9 ^b^ |
| **A119** | (*R*)-Me |  | N | CH | 2 ± 1 ^b^ | 12 ± 5 ^b^ |
