# Supplementary material for: Mycobacterium tuberculosis IMPDH in Complexes with Substrates, Products and Antitubercular Compounds
Source: PLoS One. 2015 Oct 6;10(10):e0138976. doi: 10.1371/journal.pone.0138976 (PMC4594927; doi:10.1371/journal.pone.0138976)
Supplement: S3 Table — n.d. = not determined. a. CpIMPDH data from [30]. b. Data from [25]. c. BaIMPDH data from [37]. c. Single determination. (DOCX) [file pone.0138976.s008.docx]

**S3 Table**. **SAR of enzyme inhibition for C series benzimidazole derivatives.** n.d. = not determined. a. *Cp*IMPDH data from [30]. b. Data from [25]. c. *Ba*IMPDH data from [37]. c. Single determination.

|  | | | | | |
| --- | --- | --- | --- | --- | --- |
| **Cmpd** | **R_1_** | **R_2_** | **R_3_** | ***K_i,app_* (nM)** | |
|  |  |  |  | ***Cp*IMPDH** | ***Ba*IMPDH** |
| **C11** |  | 4-CF_3_Ph | H | 220 ± 40 ^b^ | n.d. |
| **C16** |  | 4-ClPh | H | 43 ± 9 ^b^ | 120 ± 20 |
| **C61** |  | 4-ClPh | H | 30 ± 10 ^b^ | 70 ± 30 |
| **C74** |  | 4-ClPh | CH_3_ | 23 ± 4 ^b^ | n.d. |
| **C84** |  | 3,4-diClPh | H | 18 ± 5 ^b^ | 95 ± 40 |
| **C85** |  | 3,4-diClPh | H | 22 ± 5 ^b^ | n.d. |
| **C90** |  | 2-Naph | H | 7 ± 4 ^b^ | 100 ^c^ |
| **C91** |  | 2-Naph | H | 8 ± 3 ^b^ | 70 ± 20 |
| **C97** |  | 2-Naph | H | 8 ± 3 ^b^ | 15 ± 1 |
