# Supplementary material for: Mycobacterium tuberculosis IMPDH in Complexes with Substrates, Products and Antitubercular Compounds
Source: PLoS One. 2015 Oct 6;10(10):e0138976. doi: 10.1371/journal.pone.0138976 (PMC4594927; doi:10.1371/journal.pone.0138976)
Supplement: S4 Table — n.d. = not determined. a. Data from [28]. b. Data from [37]. c. Single determination. (DOCX) [file pone.0138976.s009.docx]

**S4 Table. Structures of inactive D series phthalazinone derivatives.** n.d. = not determined. a. Data from [28]. b. Data from [37]. c. Single determination.

|  | | | | |
| --- | --- | --- | --- | --- |
| **Cmpd** | **R** | **X** | ***K_i,app_* (nM)** | |
|  |  |  | **C*p*IMPDH** | ***Ba*IMPDH** |
| **D1** | 4-OMePh | H | 1000 ± 200 ^a^ | 1800 ± 30 ^b^ |
| **D20** | 3-OMePh | H | >5000 ^a^ | 11000 ^c^ |
| **D21** | 2-OMePh | H | >5000 ^a^ | n.d. |
| **D24** | 4-ClPh | H | 230 ± 50 ^a^ | 300 ± 100 ^b^ |
| **D27** | 3,4-diClPh | H | 55 ± 6 ^a^ | 280 ^c^ |
| **D29** | 4-BrPh | H | 130 ± 30 ^a^ | 80 ± 20 ^b^ |
| **D30** | 4-FPh | H | >5000 ^a^ | 7300 ^c^ |
| **D31** | 3-Cl,4-BrPh | H | 30 ± 1 ^a^ | 270 ± 100 ^b^ |
| **D41** | 2-Naphthyl | H | 61 ± 4 ^a^ | 80 ± 30 ^b^ |
| **D45** | 3-CF_3_,4-BrPh | H | 24 ± 2 ^a^ | 6500 ^c^ |
| **D48** | 3-CF_3_,4-ClPh | H | 13 ± 1 ^a^ | 4000 ± 1000 ^b^ |
| **D50** | 3-CF_3_, 4-CNPh | H | 54 ± 5 ^a^ | 1800 ± 200 |
| **D59** | 3-CF_3_, 4-BrPh | H | 32 ± 6 ^a^ | 770 ^c^ |
| **D61** |  | H | 90 ± 20 ^a^ | 290 ± 40 ^b^ |
| **D67** |  | H | 20 ± 5 ^a^ | 470 ± 10 ^b^ |
| **D69** | 3-OMe,4-ClPh | H | 31 ± 2 ^a^ | 120 ± 10 ^b^ |
| **D73** |  | H | 4 ± 1 ^a^ | 50 ± 20 ^b^ |
| **D85** | 3-OMe,4-ClPh | Me | 44 ± 3 **^b^** | 60 ± 10 ^b^ |
| **D87** |  | OH | 30 ± 10 **^b^** | 30 ± 10 ^b^ |
