# Supplementary material for: Mycobacterium tuberculosis IMPDH in Complexes with Substrates, Products and Antitubercular Compounds
Source: PLoS One. 2015 Oct 6;10(10):e0138976. doi: 10.1371/journal.pone.0138976 (PMC4594927; doi:10.1371/journal.pone.0138976)
Supplement: S5 Table — n.d. = not determined. a. Data from [26]. b. Data from [37]. (DOCX) [file pone.0138976.s010.docx]

**S5 Table. Structures of inactive P compounds.** n.d. = not determined. a. Data from [26]. b. Data from [37].

|  | | | | |
| --- | --- | --- | --- | --- |
| **Cmpd** | **X** | **R** | ***K_i,app_* (nM)** | |
|  |  |  | **C*p*IMPDH** ^a^ | ***Ba*IMPDH** |
| **P5** | CH_2_ |  | 340 ^a^ | 3000 |
| **P11** | CH_2_ | 4-Cl-Ph | 20 ± 7 ^a^ | 20 ± 1 ^b^ |
| **P12** | CH_2_ | 4-Br-Ph | 10 ± 4 ^a^ | 18 ± 4 ^b^ |
| **P13** | CH_2_ | 3,4-di-ClPh | 6 ± 1 ^a^ | 36 ± 7 ^b^ |
| **P14** | CH_2_ | 2-Naphthyl | 2.1 ± 0.8 ^a^ | 47 ± 4 ^b^ |
| **P15** | CH_2_ | 3-Cl-Ph | 70 ± 10 ^a^ | 215 ± 100 ^b^ |
| **P16** | CH_2_ | 3-CONH_2_, 4-Cl-Ph | 2.3 ± 0.8 ^a^ | 70 ± 30 ^b^ |
| **P17** | CH_2_ | 1-naphthyl | >5000 ^a^ | >5000 ^b^ |
| **P18** | CH_2_ | 3-CN, 4-Cl-Ph | 13 ± 1.6 ^a^ | 10 ± 4 ^b^ |
| **P19** | CH_2_ | 7-quinoline | 0.8 ± 0.1 ^a^ | 14 ± 2 ^b^ |
| **P20** | O | 4-Cl-Ph | 39 ± 10 ^a^ | 160 ± 60 ^b^ |
| **P22** | 3-isopropyl | 2-naphthyl | >5000 ^a^ | >5000 ^b^ |
| **P24** | O | 7-quinoline | 29 ± 18 ^a^ | 30 ± 10 ^b^ |
| **P25** | O | 3-CONH_2_, 4-Cl-Ph | 54 ± 7 ^a^ | 300 ± 100 ^b^ |
| **P26** | N-OH | 2-Naphthyl | 1.0 ± 0.2 ^a^ | 30 ± 2 ^b^ |
| **P27** | N-OH | 7-Quinoline | 0.9 ± 0.2 ^a^ | 40 ± 10 ^b^ |
| **P36** | 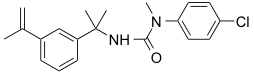 | | 900 ± 400 ^a^ | 1500 ± 700 |

|  (continued) | | | | | |
| --- | --- | --- | --- | --- | --- |
| **Cmpd** | **X** | **R** | ***K_i,app_* (nM)** | | |
|  |  |  | **C*p*IMPDH** | | ***Ba*IMPDH** |
| **P37** | N-OH | 4-Cl-Ph | 5 ± 3 ^a^ | | 20 ± 6 ^b^ |
| **P38** | CH_2_ | Ph | 250 ± 20 ^a^ | | 570 ± 120 ^b^ |
| **P39** | CH_2_ | 2-Cl-Ph | >5000 ^a^ | | >5000 ^b^ |
| **P42** | CH_2_ | 3-CONHCH_3_, 4-Cl-Ph | 7 ± 2 ^a^ | | 180 ± 90 ^b^ |
| **P47** | N-OH | 3-Cl, 4-Cl-Ph | 190 ± 10 ^a^ | | 95 ± 30 ^b^ |
| **P50** | see below | | | | |
| **P52** | see below | | | | |
| **P54** | CH_2_ | 2-Quinoline | 80 ± 20 ^a^ | | 400 ± 100 |
| **P55** | CH_2_ | 6-Quinoline | 1.8 ± 0.4 ^a^ | | 85 ± 15 ^b^ |
| **P57** | CH_2_ | 2-Quinoline | 250 ± 20 ^a^ | | 190 ± 60 ^b^ |
| **P59** | CH_2_ | 3-Quinoline | 70 ± 1 ^a^ | | 490 ± 120 ^b^ |
| **P68** | N-OH | 4-Br-Ph | 9 ± 0.4 ^b^ | | 4 ± 1 ^b^ |
| **P69** | N-OMe | 3-CN, 4-Cl-Ph | 10 ± 5 ^a^ | | 15 ± 1 ^b^ |
| **P72** | see below | | | | |
| **P74** | N-OMe | 3-CONH_2_, 4-Cl-Ph | 5 ± 2 ^a^ | | 80 ± 20 ^b^ |
| **P77** | see below | | | | |
| **P79** | see below | | | | |
| **P80** | O | 3-CF_3_, 4-Cl-Ph | | 9 ± 5 ^a^ | 30 ± 10 ^b^ |
| **P82** | N-OH | 3-CF_3_, 4-Cl-Ph | | 1.0 ± 0.1 ^a^ | 2.0 ± 0.5 ^b^ |
| **P83** | N-OMe | 3-CF_3_, 4-Cl-Ph | | 5 ± 1 ^a^ | 15 ± 2 ^b^ |
| **P93** | O | 3-NO_2_, 4-Cl-Ph | | 20 ± 10 ^a^ | 20 ± 1 ^b^ |
| **P94** | N-OH |  | | 330 ± 70 ^a^ | 100 ± 20 ^b^ |
| **P96** | N-OH | 3-NO_2_, 4-Cl-Ph | | 0.66 ± 0.08 ^a^ | 14 ± 1 ^b^ |
| **P101** | see below | | | | |
| **P102** | CH_2_ | 4-OMe-Ph | | 9 ± 1 ^a^ | 230 ± 20 ^b^ |
| **P104** | CH_2_ | 4-*t*Bu-Ph | | >5000 ^a^ | >5000 ^b^ |
| **P105** | CH_2_ | 3-OMe, 4-Cl-Ph | | 1.3 ± 0.2 ^a^ | 40 ± 10 ^b^ |
| **P106** | CH_2_ | 3-CF_3_, 4-Cl-Ph | | 4 ± 1 ^a^ | 8 ± 2 ^b^ |
| **P113** | CH_2_ |  | | 7.2 ± 0.6 ^a^ | 140 ± 50 |
| **P131** | N-OCH_2_CH_2_NH_2_ | 3-NO_2_, 4-Cl-Ph | | 20 ± 3 ^a^ |  |
