# Supplementary material for: Mycobacterium tuberculosis IMPDH in Complexes with Substrates, Products and Antitubercular Compounds
Source: PLoS One. 2015 Oct 6;10(10):e0138976. doi: 10.1371/journal.pone.0138976 (PMC4594927; doi:10.1371/journal.pone.0138976)
Supplement: S6 Table — n.d. = not determined. a. Data from [26]. b. Data from [37]. (DOCX) [file pone.0138976.s011.docx]

**S6 Table. P series: SAR of enzyme inhibition for the isopropyl and urea group.** n.d. = not determined. a. Data from [26]. b. Data from [37].

|  | | | | | | | |
| --- | --- | --- | --- | --- | --- | --- | --- |
| **Cmpd** | **R_1_** | **R_2_** | **R_3_** | **R_4_** | **X** | **IC_50_ or K_iapp_ (nM)** | |
|  |  |  |  |  |  | ***Cp*IMPDH ^a^** | ***Ba*IMPDH ^b^** |
| **P50** | 3-phenyl | Me | Me | 3-CONH_2_,4-Cl-Ph | NH | 4 ± 4 | >5000 |
| **P52** | 3-acetyl | Me | H | 4-Cl-Ph | NH | 70 ± 20 | 190 ± 50 |
| **P72** | 3-acetyl | Me | Me | 4-Cl-Ph | CH_2_ | >5000 | >5000 |
| **P77** | 3-acetyl | H | H | 4-Cl-Ph | NH | >5000 | 1200 ± 300 |
| **P79** | 4-isoprenyl | Me | H | 4-Cl-Ph | NH | 440 ± 48 | 400 ± 100 |
| **P101** |  | | | | NH | 40 ± 10 | 2400 ± 500 |
