# Supplementary material for: Mycobacterium tuberculosis IMPDH in Complexes with Substrates, Products and Antitubercular Compounds
Source: PLoS One. 2015 Oct 6;10(10):e0138976. doi: 10.1371/journal.pone.0138976 (PMC4594927; doi:10.1371/journal.pone.0138976)
Supplement: S7 Table — n.d. = not determined. n.d. = not determined a. Data from [27]. b. Data from [37]. (DOCX) [file pone.0138976.s012.docx]

**S7 Table. Structures of inactive Q compounds.** n.d. = not determined. n.d. = not determined a. Data from [27]. b. Data from [37].

|  | | | | | | |
| --- | --- | --- | --- | --- | --- | --- |
| **Cmpd** | **X** | **R_1_** | **R_2_** | **IC_50_ or K_iapp_ (nM)** | | |
|  |  |  |  | ***Cp*IMPDH ^a^** | ***Ba*IMPDH ^b^** | |
| **Q1** | Me | 2,4-di-ClPh |  | 44 ± 8 | 70 ± 4 | |
| **Q2** | Me | 2,4-di-Cl-Ph | Ph | >5000 | >5000 | |
| **Q8** | H | 2,4-di-ClPh |  | >5000 | >5000 | |
| **Q11** | Me | 1-Naphthyl |  | 9 ± 3 | 140 ± 60 | |
| **Q14** | (*S*)-Me | 1-Naphthyl |  | 26 ± 5 | 400 ± 100 | |
| **Q15** | Me | 1-Naphthyl |  | >5000 | >5000 | |
| **Q18** | Me | 4-ClPh |  | 100 ± 20 | 150 ± 10 | |
| **Q21** | (*S*)-Me | 1-Naphthyl |  | 6.1 ± 0.5 | 20 ± 4 | |
| **Q23** | (*R*)-Me | 1-Naphthyl |  | 400 ± 70 | >5000 | |
| **Q26** | (*S*)-Me | 1-Naphthyl |  | 2.7 ± 0.7 | 30 ± 6 | |
| **Q30** | Me | 1-(4-Cl-Naphthyl) |  | 28 ± 6 | 70 ± 10 | |
| **Q34** | Me | 2,6-di-ClPh |  | >5000 | >5000 | |
| **Q35** |  | | | >5000 | >5000 | |
| **Q37** |  | | | >5000 | >5000 | |
| **Q43** |  | | | >5000 | 300 ± 100 | |
| **Q44** | see below | | | | | |
| **Q45** | see below | | | | | |
| **Q48** | see below | | | | | |
| **Q52** | Me | 2-Cl, 3-NO_2_Ph |  | 49 ± 9 | | 14 ± 2 |
| **Q54** | *i*-Pr | 1-Naphthyl |  | 24 ± 2 | | 270 ± 90 |
| **Q56** |  | | | >5000 | | >5000 |
| **Q58** | (*S*)-Me | 2,3-di-Cl-Ph |  | 17 ± 6 | | 35 ± 5 |
| **Q63** |  | | | >5000 | | >5000 |
| **Q64** |  | | | 7 ± 1 | | 210 ± 10 |
| **Q81** | *i*-Pr | 2-Cl, 3-CF_3_Ph |  | 130 ± 20 | | 140 ± 5 |

| **** | | | | |
| --- | --- | --- | --- | --- |
| **Cmpd** | **R_1_** | **X** | ***K_iapp_* (nM)** | |
|  |  |  | ***Cp*IMPDH ^a^** | ***Ba*IMPDH ^b^** |
| **Q44** | Me | CH_2_Ph | > 5000 | > 5000 |
| **Q45** | Me | Phenyl | 120 ± 20 | 200 ± 100 |
| **Q48** | (*S*)-Me | Phenyl | > 5000 | 900 ± 200 |
