# Supplementary material for: Mycobacterium tuberculosis IMPDH in Complexes with Substrates, Products and Antitubercular Compounds
Source: PLoS One. 2015 Oct 6;10(10):e0138976. doi: 10.1371/journal.pone.0138976 (PMC4594927; doi:10.1371/journal.pone.0138976)
Supplement: S8 Table — n.d. = not determined. a. Data from [26]. b. Data from [27]. c. Data from [37]. d. Single determination. e. Two determinations. (DOCX) [file pone.0138976.s013.docx]

**S8 Table. *Cp*IMPDH inhibitors with antitubercular activity: comparison of enzyme inhibition.** n.d. = not determined. a. Data from [26]. b. Data from [27]. c. Data from [37]. d. Single determination. e. Two determinations.

| **Cmpd** | ***Cp*IMPDH** | ***M. tuberculosis*** | | ***B. anthracis*** | |
| --- | --- | --- | --- | --- | --- |
|  | ***K_iapp_* (nM)** | ***K*_i,app_ (nM)** | **MIC (μM)** | ***K*_i,app_ (nM)** | **MIC (μM)** |
| **P32** | 5 ± 1 ^a^ | 158 ^d^ | 11.4 | 50 ± 5 ^c^ | 25 |
| **P41** | 7.6 ± 1.3 | 17 ± 4 | 5.0 | 37 ± 23 | 25 |
| **P67** | 4.2 ± 0.8 ^c^ | 13 ± 5 | 2.9 | 40 ± 20 ^c^ | 23 |
| **P146** | 30 ± 10 ^c^ | 37 ± 8 | 14.9 | 170 ± 10 ^c^ | 1 |
| **P150** | 25 ± 10 ^c^ | 35 ± 3 | 14.2 | 40 ± 20 ^c^ | 0.5 |
| **Q9** | 40 ± 5 ^b^ | 650 ± 140 | 17.5 | 500 ± 200 ^c^ | >50 |
| **Q22** | 210 ± 20 ^b^ | 2000 ± 60 ^c^ | 19.8 | n.d. | n.d. |
| **Q27** | 19 ± 2 | 240 ± 40 | 15.2 | 260 ± 20 | >50 |
| **Q33** | 28 ± 2 | 150 ± 50 | 5.3 | 37 ± 9 | >50 |
| **Q36** | 1.2 ± 0.2 | 76 ± 27 ^c^ | 9.4 | 10 ± 1 | n.d. |
| **Q42** | 50 ± 10 | 620 ± 70 | 17.3 | 75 ± 1 | n.d. |
| **Q46** | 2.3 ± 0.9 | 130 ± 40 | 11.6 | 12 ± 5 | >50 |
| **Q49** | 22 ± 6 | 440 ± 40 | 12.7 | 400 ± 100 | >50 |
| **Q59** | 0.6 ± 0.5 | 40 ± 7 | 9.7 | 10 ± 3 | >50 |
| **Q60** | 2 ± 1 ^b^ | 40 ± 16 | 14.5 | 13 ± 1 ^c^ | nd |
| **Q67** | 0.5 ± 0.1 ^b^ | 14 ± 3 | 6.7 | 5 ± 1 ^c^ | 12 |
| **Q77** | 13 ^d^ | 100 ± 20 | 6.3 | 130 ± 20 | nd |
