# Supplementary material for: Mycobacterium tuberculosis IMPDH in Complexes with Substrates, Products and Antitubercular Compounds
Source: PLoS One. 2015 Oct 6;10(10):e0138976. doi: 10.1371/journal.pone.0138976 (PMC4594927; doi:10.1371/journal.pone.0138976)
Supplement: S9 Table — * Indicates tight binding conditions. C, competitive inhibition; UC, uncompetitive inhibition, NC, noncompetitive inhibition. (DOCX) [file pone.0138976.s014.docx]

**S9 Table. Inhibition of *Mtb*IMPDH2ΔCBS**. * Indicates tight binding conditions. C, competitive inhibition; UC, uncompetitive inhibition, NC, noncompetitive inhibition.

|  | **MAD1** | **P41 *** | **Q67 *** |
| --- | --- | --- | --- |
| *K*_i,app_ (nM) | 1580 ± 70 | 21 ± 3 | 14 ± 3 |
| *K*_i_ vs. IMP (nM) | 1000 ± 50 (UC) | 9 ± 4 (UC) | 17 ± 9 (UC) |
| *K*_i_ vs. NAD^+^ (nM) | 1400 ± 100 (UC) | 13 ± 4 (NC) | 7 ± 7 (C) |
